# A Hydrogen Sulfide–Releasing Dynamic Hydrogel Modulates Coordinated Neurovascular, Immune, and Angiogenic Responses for Scar‐Suppressed Diabetic Wound Repair

**DOI:** 10.1002/advs.76337

**Published:** 2026-06-27

**Authors:** Xuyang Ning, Ziqiang Zhou, Bangming Li, Hong Lu, Haoyang Wen, Zhoulong Huang, Gang Li, Ping Hu

**Affiliations:** ^1^ Department of Burns & Plastic Surgery Faculty of Medical Science Guangzhou Red Cross Hospital Jinan University Guangzhou China; ^2^ State Key Laboratory of Bioactive Molecules and Druggability Assessment Jinan University Guangzhou China; ^3^ College of Pharmacy Jinan University Guangzhou China; ^4^ First Clinical Medical College Guangdong Medical University Zhanjiang China

**Keywords:** diabetic wound healing, dynamic hydrogel, hydrogen sulfide donor, macrophage modulating, neural regeneration, therapeutic angiogenesis

## Abstract

Diabetic wound healing is impaired by a pathological microenvironment involving immune dysregulation, deficient angiogenesis, and compromised neural repair. Here, we engineered a dynamic phenylboronate ester–crosslinked hyaluronic acid/poly(vinyl alcohol) (HA/PVA) hydrogel for spatiotemporally controlled delivery of a novel hydrogen sulfide (H_2_S) donor. In diabetic models, this hydrogel functions as both a reactive oxygen species (ROS) scavenger and an immunomodulatory platform. Sustained H_2_S release promotes macrophage polarization toward the pro‐reparative M2 phenotype, enhances angiogenesis through activation of the vascular endothelial growth factor (VEGF) signaling pathway, and facilitates sensory nerve repair by restoring calcitonin gene–related peptide (CGRP) and nerve growth factor (NGF) levels. Transcriptomic analysis further reveals that H_2_S regulates gene networks associated with antioxidant defense, immune modulation, angiogenesis, and neuroprotection. In a diabetic rabbit ear model, this treatment markedly accelerates wound closure and suppresses hypertrophic scar formation by regulating collagen deposition and remodeling. Mechanistically, H_2_S inhibits fibrosis through suppression of the transforming growth factor‐β (TGF‐β)/Smad signaling pathway. Collectively, this work presents an integrated therapeutic strategy that coordinates immune, vascular, and neural repair processes, offering a promising approach for diabetic wound regeneration and scar mitigation.

## Graphical abstract

ROS‐Responsive H_2_S Hydrogel (HAPPF) Coordinated Regeneration for High‐Quality Diabetic Wound Repair.A self‐regulating dynamic hydrogel is developed to deliver a fluorogenic H_2_S donor in response to excessive ROS. Released H_2_S acts as a master regulator to resolve chronic inflammation (M2 polarization), restore VEGF‐driven angiogenesis, and rebalance neuro‐immune signaling (CGRP/NGF). This orchestrated multidimensional repair network suppresses TGF‐β‐driven fibrotic pathways (TGF‐β/Smad), collectively revitalizing the pathologically arrested diabetic wound toward rapid healing with markedly reduced scarring and improved tissue architecture. This system establishes localized, stimulus‐responsive gaseous signaling as a central orchestrator for comprehensive, functional tissue repair.

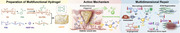

## 1 Introduction

The management of diabetic wounds poses a formidable global clinical challenge and an escalating socioeconomic burden [1]. These wounds exist in a pathologically arrested state, trapped in a self‐perpetuating vicious cycle of chronic inflammation, pervasive oxidative stress, impaired angiogenesis, and dysregulated innervation [2, 3, 4, 5]. This dysfunctional microenvironment fails to progress through the orderly phases of healing, culminating in a high risk of infection, ulceration, and ultimately, amputations.

At the epicenter of this pathology lies persistent oxidative stress. The overproduction of ROS acts as a master switch, not only inflicting direct cellular damage but also perpetuating the cycle by hyperactivating the nuclear factor kappa‐light‐chain‐enhancer of activated B cells (NF‐κB) pathway [6]. This fuels a pro‐inflammatory feedback loop, characterized by sustained elevation of cytokines like tumor necrosis factor‐alpha (TNF‐α), which concurrently damages endothelial cells and stifles pro‐angiogenic signaling [7, 8]. This inflammatory milieu critically disrupts immune homeostasis, notably impairing the crucial phenotypic switch of macrophages from pro‐inflammatory (M1) to pro‐repair (M2) states—a transition pivotal for initiating angiogenesis [9, 10, 11]. Consequently, a hallmark of diabetic wounds is the failure of angiogenesis, rooted in the compromised expression and signaling of key growth factors like VEGF [12].

Compounding this, diabetic neuropathy disrupts the neuro‐immune‐vascular axis, creating a deficiency in neuro‐modulators such as the potent vasodilator and immunomodulator CGRP, alongside an imbalance in NGF [13, 14, 15]. Recent studies have further highlighted that CGRP released from sensory nerve fibers not only regulates vascular tone and endothelial function, but also directly modulates immune cell activity, thereby serving as a critical neuro‐immune interface in wound repair. Importantly, CGRP has been shown to interact functionally with angiogenic and fibrotic pathways, where it can enhance VEGF‐mediated angiogenesis while concurrently suppressing excessive TGF‐β–driven fibrotic responses, thereby coordinating vascular regeneration and scar formation control during tissue repair. This establishes CGRP as a pivotal signaling mediator linking neural activity with both angiogenic and fibrotic regulation in the wound microenvironment [16].

This triad of sustained inflammation, defective angiogenesis, and impaired neuro‐immune crosstalk collectively undermines coordinated tissue repair, rendering diabetic wounds highly refractory to conventional therapies [17, 18, 19]. Despite advances in wound care, current strategies often offer only symptomatic relief and fall short of fundamentally modulating this pathological milieu toward regeneration.

The gaseous signaling molecule H_2_S has recently emerged as a key regulator of tissue repair, demonstrating efficacy in mitigating oxidative stress, resolving inflammation, and promoting angiogenesis, notably through VEGF upregulation [20, 21, 22]. Its capability to modulate core signaling pathways, including activation of the nuclear factor erythroid 2–related factor 2/antioxidant response element (Nrf2/ARE) axis and suppression of NF‐κB, positions H_2_S as an attractive candidate for interrupting the pathological cycle of diabetic wounds [20, 23, 24, 25]. However, the clinical translation of H_2_S remains severely constrained by its intrinsic physicochemical limitations, including a short half‐life, poor targeting, and a narrow therapeutic window. These challenges necessitate a delivery system capable of achieving localized, sustained, and stimulus‐responsive H_2_S release at the wound site.

In this context, smart hydrogel dressings have garnered increasing attention. Among them, hydrogels crosslinked via dynamic boronate ester bonds, formed between 3‐aminophenylboronic acid (PBA) modified hyaluronic acid and polyvinyl alcohol (PVA), offer unique advantages. These dynamic bonds are inherently ROS‐responsive, enabling the hydrogel to function not merely as a passive barrier but as an active therapeutic matrix capable of scavenging excessive ROS [26, 27, 28]. Importantly, ROS‐triggered bond dissociation facilitates on‐demand hydrogel remodeling and drug release. We therefore hypothesized that integrating a ROS‐responsive H_2_S donor into such a dynamic matrix would create a closed‐loop, self‐regulating therapeutic system, in which the pathological stimulus (ROS) autonomously governs the release of the therapeutic mediator (H_2_S).

**SCHEME 1:**
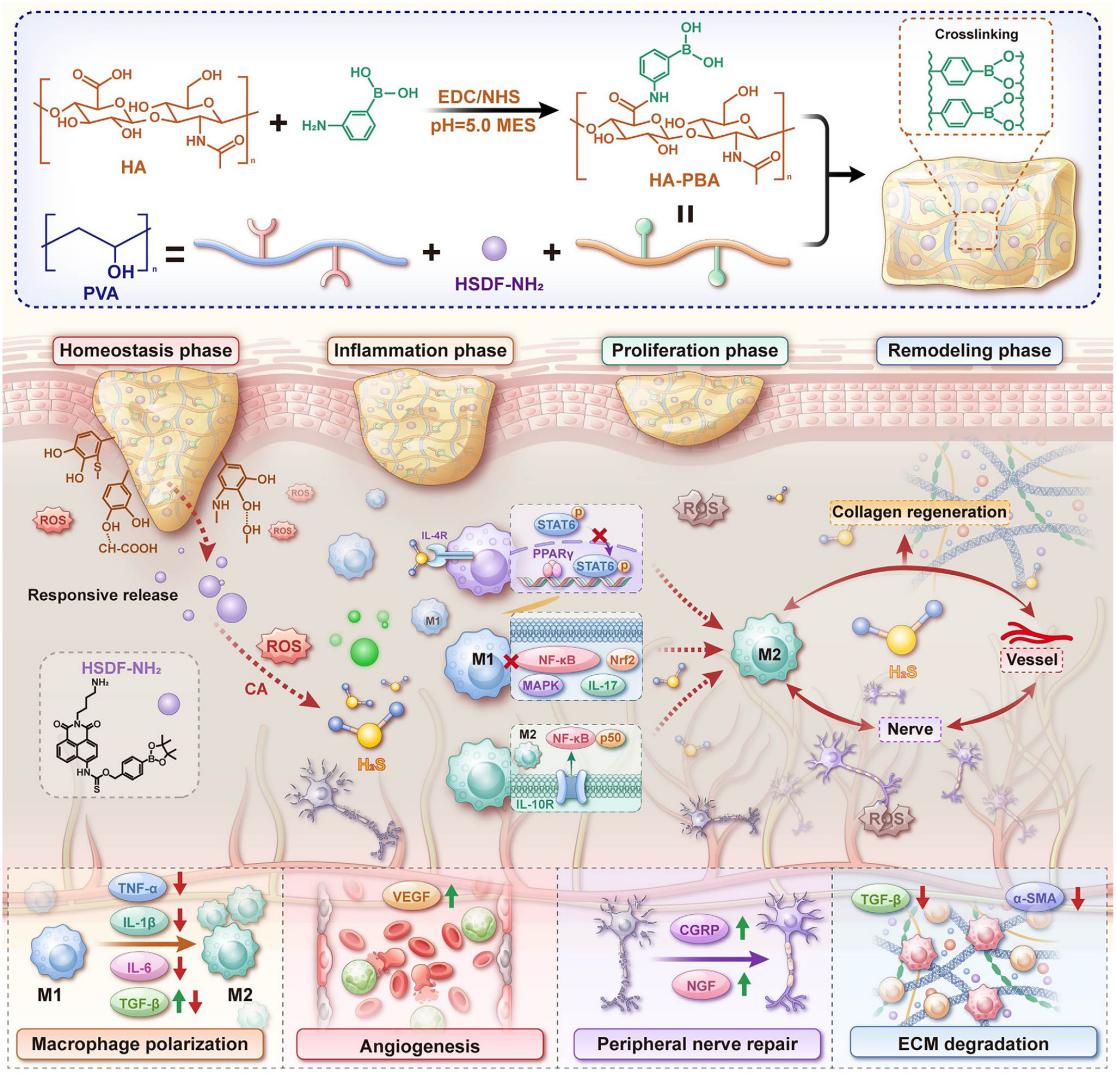
Mechanism of the H_2_S‐releasing dynamic hydrogel (HAPPF) for modulating the diabetic wound microenvironment. The hydrogel is formed by crosslinking PBA‐HA and PVA, and is loaded with the ROS‐responsive H_2_S donor HSDF‐NH_2_. In the diabetic wound microenvironment, the hydrogel scavenges excessive ROS, leading to dynamic network dissociation and the controlled release of H_2_S. The released H_2_S then coordinately suppresses key pro‐inflammatory factors (TNF‐α, IL‐6, IL‐1β, and TGF‐β1), promotes macrophage M2 polarization, drives VEGF‐mediated angiogenesis, and restores the balance of neuroregulatory factors (CGRP/NGF), thereby breaking the pathological cycle and enabling scar‐suppressed wound healing.

Building upon this concept, we incorporated HSDF‐NH_2_, a H_2_S donor previously developed by our group, into a PBA‐HA/PVA dynamic hydrogel (denoted as HAPPF). HSDF‐NH_2_ is specifically engineered to respond to elevated ROS levels, releasing H_2_S while simultaneously generating a green fluorescence signal that enables real‐time tracking of donor activation [29, 30]. This donor has been rigorously validated in models of brain injury, nerve repair, and cutaneous wound healing, demonstrating potent cytoprotective and pro‐regenerative effects [30, 31, 32]. We hypothesize that encapsulating HSDF‐NH_2_ within the PBA‐HA/PVA hydrogel (HAPPF) would establish a dual ROS‐scavenging and H_2_S ‐release system capable of sustained, pathology‐activated therapeutic signaling. We further postulate that the released H_2_S will function as a central regulator regulating a comprehensive repair program by simultaneously alleviating oxidative stress and inflammation, promoting macrophage M2 polarization, restoring VEGF‐driven angiogenesis, and rebalancing the neuro‐immune milieu through CGRP and NGF signaling, thereby revitalizing the antioxidant‐immune‐angiogenic‐neural axis (Scheme 1).

To validate these hypotheses, we systematically evaluated the therapeutic performance of HAPPF. The hydrogel exhibited potent ROS–scavenging capacity, effectively induced macrophage polarization, and promoted endothelial tubulogenesis, accompanied by upregulated expression of VEGF, CGRP, and NGF. In a diabetic rat full‐thickness wound model, HAPPF significantly accelerated wound closure and supported high‐quality tissue regeneration. Transcriptomic and mechanistic analyses further revealed that these reparative effects were mediated through the coordinated regulation of immune, angiogenic, and neuro‐regenerative pathways. Notably, transcriptomic profiling suggested that the anti‐scar effects of HAPPF involved modulation of PPARγ, IL‐17, and NF‐κB signaling pathways, along with downregulation of profibrotic mediators such as TGF‐β and α‐smooth muscle actin (α‐SMA). Importantly, in a clinically relevant diabetic rabbit ear model, we further demonstrated that HAPPF‐mediated H_2_S release significantly attenuated fibrotic scar formation, which was closely associated with suppression of the TGF‐β/Smad signaling pathway, as evidenced by reduced expression of TGF‐β, p‐Smad2, and p‐Smad3. Consistently, in a clinically relevant diabetic rabbit ear model, HAPPF not only accelerated wound healing but also markedly suppressed fibrotic scar formation, thereby validating these mechanistic insights. However, we also acknowledge that these conclusions are primarily based on multi‐level correlative evidence (histological, transcriptomic, and biochemical analyses). Although these data collectively support the proposed mechanisms, direct pathway‐specific intervention studies are still required in future work to fully establish causal relationships.Collectively, this study establishes a biorthogonal HSDF‐NH_2_–driven H_2_S delivery platform as an effective strategy for microenvironmental modulating, positioning H_2_S as a central orchestrator of multidimensional regeneration in diabetic wound repair.

## 2 Results and Discussion

### 2.1 Synthesis and Characterization of HAPP

**FIGURE 1:**
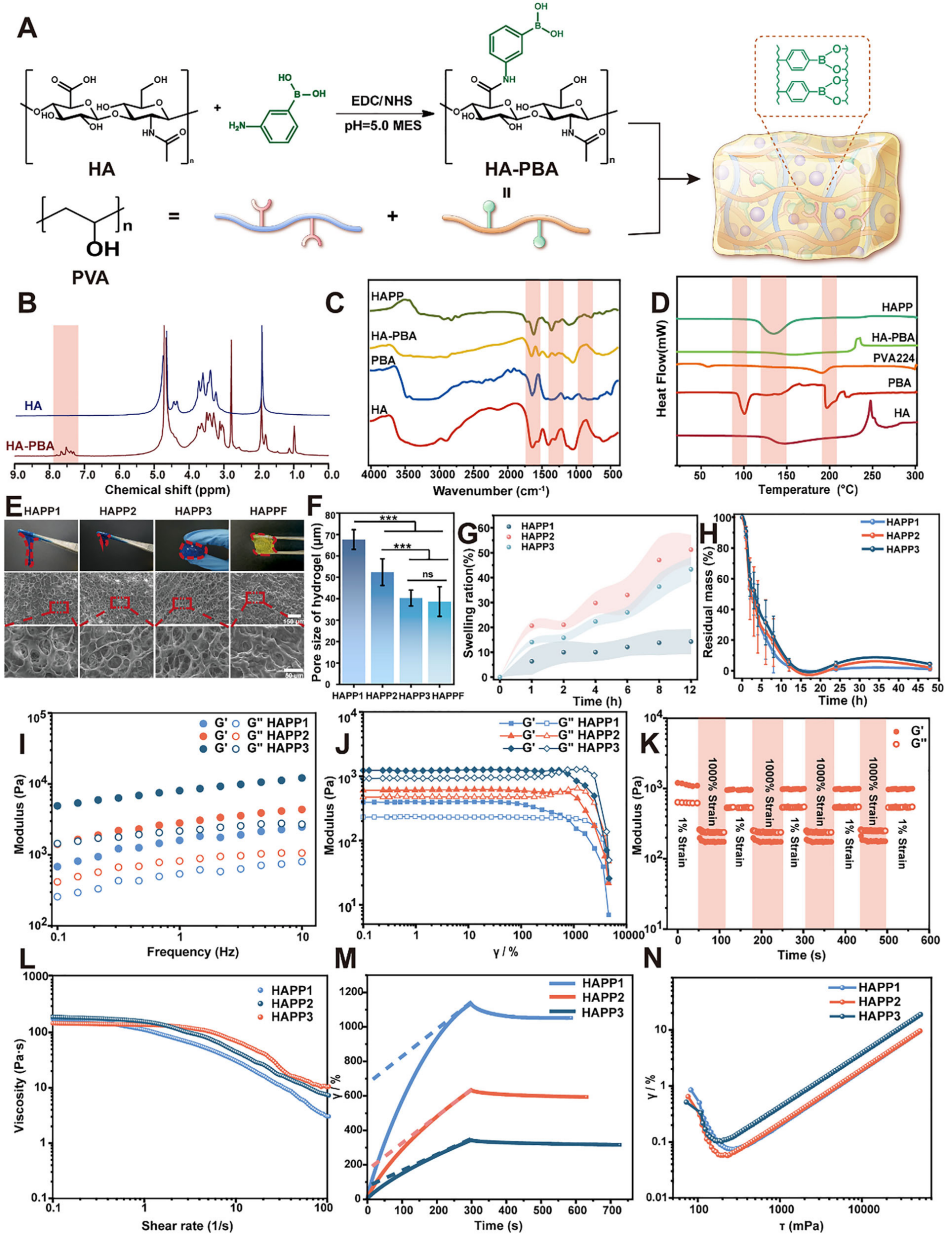
Synthesis and characterization of HAPP hydrogels. (A) Schematic illustration of the synthesis of HA‐PBA and the boronate ester‐based crosslinking mechanism forming the HAPP hydrogel network.(B) ^1^H NMR spectra of HA and HA‐PBA confirming successful PBA grafting.(C) FTIR spectra of HA, PBA, HA‐PBA, and HAPP showing characteristic peaks of boronate ester bonds.(D) DSC thermograms of HA, PBA, PVA224, HA‐PBA, and HAPP, revealing thermal transitions and enhanced molecular interactions after gel formation.(E) Macroscopic images and SEM micrographs of HAPP hydrogels with different PVA224 contents, showing porous networks with tunable pore sizes.(F) Statistical analysis of the pore sizes(*n* = 20 independent samples).(G)Swelling behavior of HAPP hydrogels in PBS at 37°C(*n* = 3 independent samples).(H) Moisture retention performance under 30% relative humidity, indicating their capacity to maintain a moist environment(*n* = 3 independent samples).(I) Frequency sweep curves (0.1–10 Hz, 1% strain, 37°C) demonstrating viscoelastic behavior of HAPP hydrogels.(J) Linear viscoelastic region (LVR) scan showing the stability of the network structure under small deformations.(K) Step‐strain sweep (1% and 1000%, 1 Hz, 37°C) showing reversible recovery behavior and self‐healing capability.(LK) Shear‐thinning curves indicating injectability of HAPP.(M) Creep‐recovery tests confirming reversible deformation and network recovery.(N) Yield stress analysis showing mechanical robustness and resilience of the hydrogel network (^***^ *p* < 0.001, ns = no significance by one‐way ANOVA followed by Tukey's post hoc test).

HA‐PBA was synthesized via an amidation reaction between HA and PBA under mildly acidic conditions. The obtained HA‐PBA was then crosslinked with PVA224 through dynamic boronate ester bonds to form the HAPP hydrogel network (Figure 1A). The ^1^H NMR spectrum of HA‐PBA exhibited new aromatic proton peaks at 7.53 ppm (m, 4H) and 1.80 ppm (s, 3H), which were absent in HA, confirming the successful grafting of PBA. The characteristic peak at 7–8 ppm corresponded to protons on the benzene ring, and the grafting ratio of PBA was calculated to be approximately 41.8% (Figure 1B) [33]. FTIR spectra revealed characteristic peaks of HA‐PBA at 700–980 cm^−^^1^ (B─C stretching) and 1280–1340 cm^−^^1^ (B─O vibration), while HAPP displayed a distinct B─O stretching peak at ∼1380 cm^−^^1^, along with a weakened ─OH band at 3000–3500 cm^−1^, confirming the formation of boronate ester crosslinks (Figure 1C). Differential scanning calorimetry (DSC) analysis further confirmed structural transitions during synthesis. The endothermic peak of HA appeared at 245.8°C, which shifted to 231.3°C for HA‐PBA due to partial disruption of crystalline domains by PBA grafting. Notably, after forming the HAPP hydrogel (HAPP2), only a single broad exothermic peak was observed at 133.2°C, distinct from both PVA224 and HA‐PBA (Figure 1D). This transformation suggests the establishment of a homogeneous dynamic boronate ester network, accompanied by the disappearance of individual polymer crystalline transitions, indicative of strong molecular interactions and uniform crosslinking.

Hydrogels prepared with 3 wt.% HA‐PBA and varying PVA224 concentrations (HAPP1: 2%, HAPP2: 3%, HAPP3: 4%) displayed distinct porous morphologies under SEM. As the PVA224 content increased, the pore size decreased from 75.22 ± 6.47 µm (HAPP1) to 51.50 ± 7.57 µm (HAPP3), reflecting a higher crosslinking density. Macroscopically, these hydrogels exhibited reduced viscous behavior and enhanced solid‐like characteristics (Figure 1E,F). Upon the incorporation of HSDF‐NH_2_ into the HAPP2 system, the resulting drug‐loaded hydrogel (HAPPF) maintained a well‐organized porous structure; however, the pore walls became significantly thicker, likely due to drug loading or local recrystallization induced by solvent evaporation. The average pore size of HAPPF further decreased to 38.65 ± 0.89 µm, suggesting that the interaction between the drug and the polymer skeleton, combined with the solvent effect of DMSO, promoted the densification of the network—a structural feature conducive to achieving controlled drug release. Under highly hydrated conditions, all hydrogels showed moderate swelling capability, effectively absorbing wound exudates while maintaining morphological stability (Figure 1G). In a dry environment (relative humidity ∼30%), they sustained a moist microenvironment for approximately 6 h. The swelling ratio initially increased and then decreased with increasing PVA224 content, while moisture retention showed minimal variation among samples (Figure 1H). Notably, HAPP2 achieved a balanced swelling ratio and hydration retention (51% swelling and 25% retention after 6 h), reflecting its superior ability to maintain a stable yet flexible network suitable for wound healing applications.

Considering the irregular geometry of wounds and the dynamic mechanical strain induced by skin motion, the viscoelastic and adhesive performances of HAPP hydrogels were further evaluated. As shown in Figure 1I, the storage modulus (G′) of all samples remained higher than the loss modulus (G″) across 0.1–10 Hz, confirming their elastic‐dominant nature. Both G′ and G″ increased significantly with rising PVA224 content, consistent with a denser crosslinked network. Among them, HAPP2 exhibited an optimal G′ of ∼2 kPa at 37°C and 1 Hz, closely matching the viscoelastic modulus range of native human skin, thereby providing sufficient elasticity and compliance while minimizing discomfort or foreign‐body sensation [34].

Dynamic strain sweep tests revealed typical shear‐thinning behavior, where both G′ and G″ decreased progressively with strain amplitude, and a crossover point (G″ > G′) appeared at 551.2%, 671%, and 644% for HAPP1, HAPP2, and HAPP3, respectively(Figure 1J). The higher crossover strain of HAPP2 indicates its enhanced tolerance to deformation before yielding, implying a balanced network stability and flexibility. When the strain returned to 1%, G′ recovered immediately to its initial value, and this recovery was fully reversible over multiple cycles, demonstrating excellent injectability and self‐healing ability (Figure 1K,L).

Creep–recovery and yield stress measurements further validated these findings. Under constant stress, HAPP1, HAPP2, and HAPP3 exhibited elastic deformations of 692.2%, 196.9%, and 87.97%, with recoverable strains of 83.70%, 40.50%, and 27.83%, respectively. The higher recovery rate of HAPP2 suggests an optimal dynamic equilibrium between reversible boronate ester bonds and hydrogen bonding interactions(Figure 1M). Yield stress increased from 161.8 Pa (HAPP1) to 243.6 Pa (HAPP3), implying improved resistance to flow deformation with higher polymer concentration(Figure 1N). Structural recovery analysis revealed that HAPP2 required 4.58 s to achieve 85% recovery and displayed a recovery ratio of 49.57% after 1 min, corresponding to an absolute change of 90.23 Pa·s, indicating rapid and efficient network reconstruction compared with HAPP1 and HAPP3(Figure S1).

Taken together, HAPP2 achieved a favorable balance between mechanical strength, flexibility, and viscoelastic stability. Its skin‐compliant modulus, fast self‐recovery, and stable hydration behavior make it particularly suitable as a self‐healing adhesive hydrogel dressing for dynamic wound environments.

### 2.2 Self‐Healing and Environment‐Responsive H_2_S/Fluorescence Release of HAPPF Hydrogels

**FIGURE 2:**
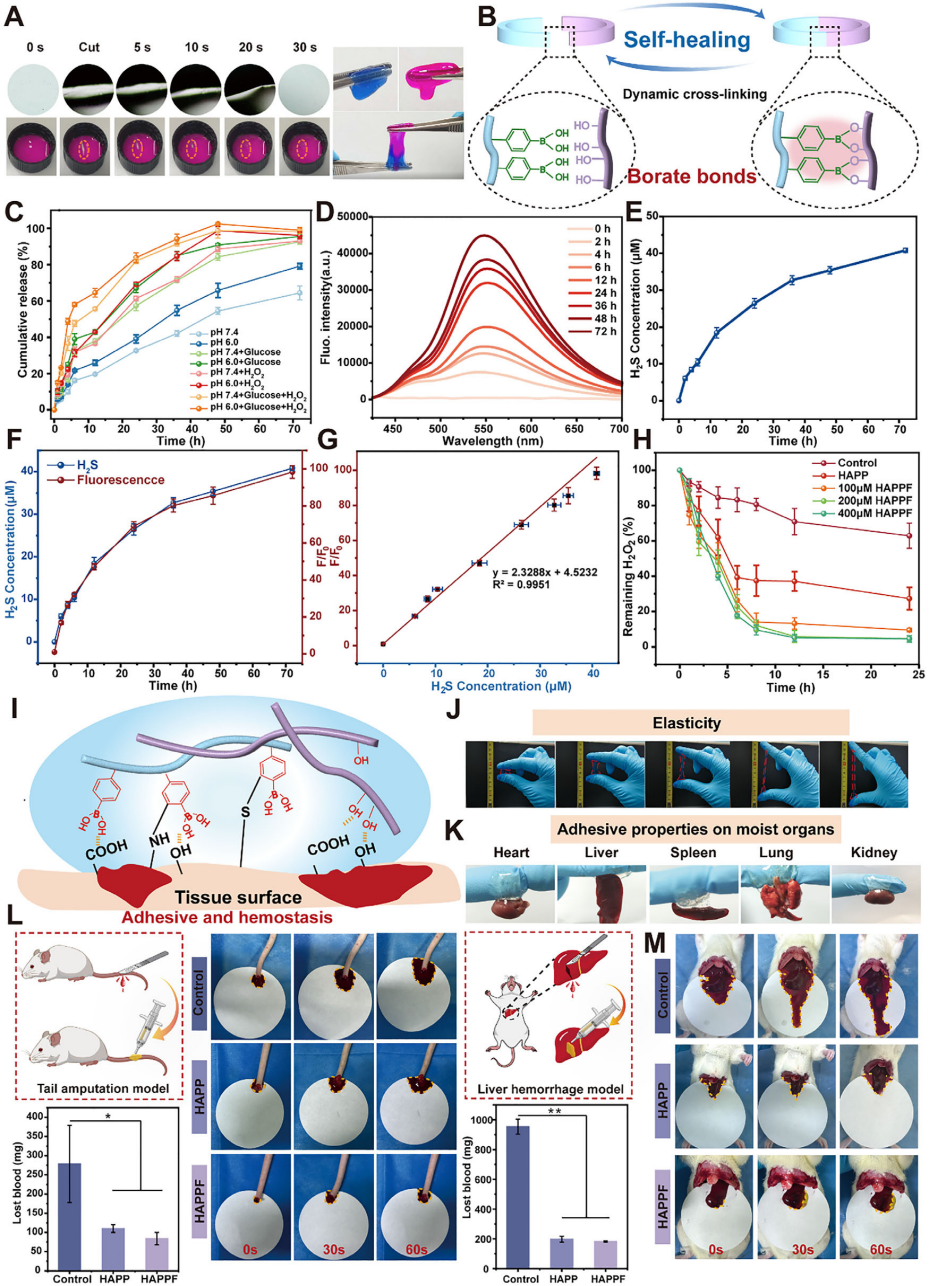
Multifunctional characterization of HAPPF hydrogels. (A) Macroscopic and microscopic images demonstrating seamless self‐healing of HAPPF hydrogels and interface fissure disappearance within 30 s.(B) Schematic of dynamic boronate ester and hydrogen bond interactions enabling reversible self‐repair.(C) Cumulative HSDF‐ NH_2_ release under different environmental conditions.(D) Time‐dependent fluorescence emission of HAPPF over 72 h, demonstrating intrinsic self‐reporting behavior.(E) Kinetics of H_2_S generation quantified by methylene blue assay.(F) Fluorescence change over time reflecting H_2_S release.(G) Correlation between fluorescence intensity and H_2_S release.(H) H_2_O_2_ scavenging efficiency of HAPPF and HAPP hydrogels.(I) Schematic of tissue adhesion, highlighting PBA interactions with hydroxyl, carboxyl, and amino groups.(J) Macroscopic tensile test showing hydrogel stretchability to ∼9 cm.(K) Adhesion on moist ex vivo tissues (heart, liver, spleen, lung, kidney).(L) Tail‐transection hemostasis assay in rats, showing rapid bleeding arrest within 60 s.(M) Liver hemorrhage model demonstrating effective blood loss reduction (*n* = 3 independent samples, ^*^ *p* < 0.05, ^**^ *p* < 0.01, ^***^ *p* < 0.001 by one‐way ANOVA followed by Tukey's post hoc test).

HAPPF hydrogels exhibit exceptional autonomous self‐healing via dynamic boronate ester bonds and synergistic hydrogen bonding. Macroscopically, dye‐labeled hydrogel segments (Rhodamine B/Methylene Blue) merged seamlessly upon contact without external stimuli. Both macroscopic photography and optical microscopy confirmed that mechanical fissures completely disappeared within 30 s at room temperature, restoring structural integrity (Figure 2A,B). This rapid, reversible repair—driven by the dynamic dissociation and re‐association of HA‐PBA and PVA chains—ensures functional stability and compliance during complex skin movement.

In drug release experiments, HSDF‐NH_2_ exhibited a cumulative release of approximately 50% over 48 h in neutral PBS (pH *7.4*), indicating stable encapsulation. Acidic pH, glucose, or H_2_O_2_ accelerated release, with nearly 100% release achieved within 48 h under simulated diabetic wound conditions (pH *6.0*, 5 mg/mL glucose, 100 mm H_2_O_2_) (Figure 2C). This acceleration arises from H_2_O_2_‐induced oxidation of boronate ester bonds and competitive glucose binding, which relaxes the hydrogel network and facilitates molecular diffusion.

Notably, released HSDF‐NH_2_ reacts with H_2_O_2_ and carbonic anhydrase (CA) to generate H_2_S accompanied by self‐reporting fluorescence. Fluorescence intensity progressively increased over 72 h, reaching approximately 100‐fold higher than the initial level (Figure 2D), serving as an internal reference for normalized analysis of H_2_S release. Quantification using the methylene blue assay revealed that 200 µm HSDF‐NH_2_ produced ∼40.82 µm H_2_S within 72 h (Figure 2E). Fluorescence intensity closely correlated with H_2_S generation (R^2^ = 0.9951, Figure 2F,G), demonstrating that fluorescence can serve as a real‐time, visual indicator for quantitative monitoring of H_2_S release.

Furthermore, both HAPPF and HAPP hydrogels exhibited potent H_2_O_2_ scavenging capabilities. In the presence of 200 µM HSDF‐NH_2_, approximately 52% of H_2_O_2_ was eliminated within 4 h, significantly higher than that of HAPP without HSDF‐NH_2_ (38%) (Figure 2H). Notably, HAPPF achieved nearly complete removal of H_2_O_2_ within 12 h (Figure S2), indicating its superior and sustained antioxidant performance. This enhanced ROS‐scavenging ability can be attributed to the synergistic effects between the controlled H_2_S release from HSDF‐NH_2_ and the ROS‐responsive boronate ester linkages, which together alleviate oxidative stress and establish a favorable microenvironment for tissue regeneration.

### 2.3 Tissue Adhesion and Hemostatic Performance of HAPPF Hydrogels

The remarkable tissue adhesion of HAPPF hydrogels is primarily attributed to the dynamic covalent and non‐covalent interactions between PBA moieties and functional groups on the skin, including carboxyl, amino, and hydroxyl residues of glycosaminoglycans and epidermal proteins. PBA forms reversible boronate ester bonds with hydroxyl groups on the tissue surface, while its vacant orbital structure facilitates additional electrostatic and hydrogen‐bonding interactions with carboxyl and amino groups, collectively enhancing hydrogel adhesion under wet conditions. The hydroxyl groups of PVA chains further stabilize the interfacial attachment via hydrogen bonding, providing compliance that allows the hydrogel to accommodate subtle skin curvature and movement without detachment (Figure 2I).

Macroscopic tensile tests demonstrated that HAPPF hydrogels can be stretched up to approximately 9 cm at room temperature, reflecting excellent extensibility and flexibility(Figure 2J). This performance is ascribed to the three‐dimensional dynamic crosslinked network and the reversible breakage and reformation of PBA‐PVA interactions, which dissipate applied strain and enable rapid structural recovery during stretching. In ex vivo adhesion tests on moist organ surfaces (heart, liver, spleen, lung, and kidney), HAPPF hydrogels adhered firmly and remained attached even under gentle tilting or tissue movement, demonstrating their adaptability to complex physiological environments (Figure 2K).

Regarding hemostatic performance, HAPPF hydrogels rapidly arrested bleeding in a tail‐transection model, achieving hemostasis within 60 s and reducing blood loss to one‐third of the control group (Figure 2L). In a liver hemorrhage model, application of the hydrogel reduced blood loss by approximately 771.25 mg compared with untreated controls (Figure 2M). The observed rapid hemostasis is primarily attributed to strong adhesion and sealing at the bleeding site, while the three‐dimensional network structure facilitates blood absorption and concentrates platelets and coagulation factors, thereby promoting local clot formation.

### 2.4 Biocompatibility, Migration Enhancement, and Angiogenic Potential of HAPPF Hydrogels

**FIGURE 3:**
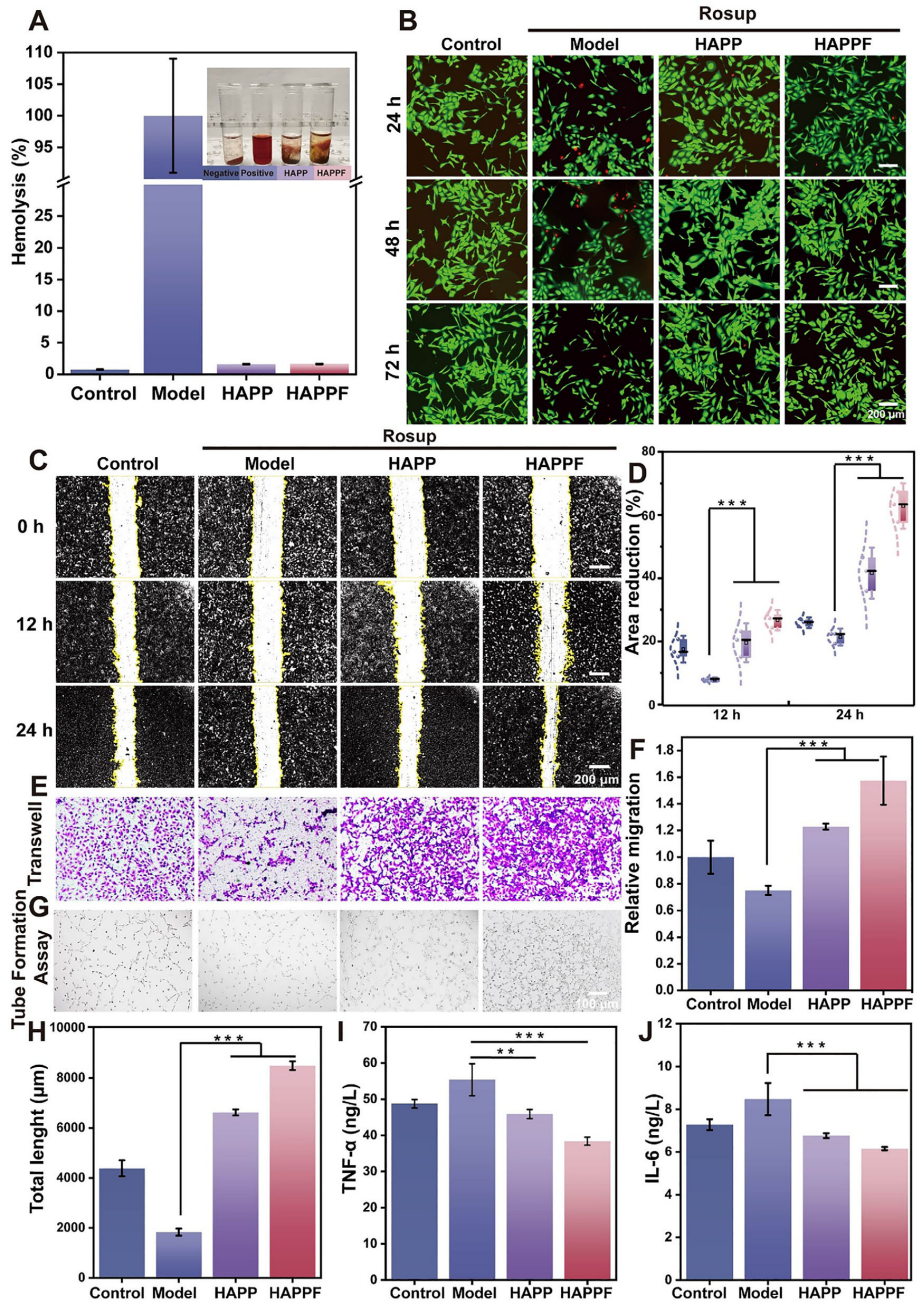
Evaluation of the biocompatibility, migration, and angiogenesis of HAPPF hydrogels. (A) Hemolysis assay of HAPP and HAPPF hydrogels, with inset showing red blood cells incubated with hydrogels. (B) Live/dead staining images of HUVECs cultured with hydrogel extracts for 24, 48, and 72 h (Scale bar: 200 µm). (C) Cell migration images of HUVECs after incubation with hydrogels for 0, 12, and 24 h (Scale bar: 200 µm). (D) Quantitative analysis of migration areas at 12 and 24 h for different groups. (E) Transwell staining images of HUVECs after 12 h incubation with hydrogels (Scale bar: 200 µm). (F) Quantitative analysis of migrated cells stained with crystal violet. (G) Tube formation of HUVECs after 6 h incubation with hydrogels (Scale bar: 100 µm). (H) Quantification of total tube length. (I,J) Enzyme‐Linked Immunosorbent Assay (ELISA) measurement of TNF‐α and IL‐6 expression in HUVECs under different treatments. (*n* = 3 independent samples, ^*^ *p* < 0.05, ^**^ *p* < 0.01, ^***^ *p* < 0.001 by one‐way ANOVA followed by Tukey's post hoc test).

Human umbilical vein endothelial cells (HUVECs) were employed to evaluate the biocompatibility, migration‐promoting effects, and angiogenic potential of HAPPF and HAPP hydrogels. Hemocompatibility was first assessed by hemolysis testing. As shown in Figure 3A, the hemolysis rate of HAPPF hydrogel was 1.63% (HAPP: 1.58%), far below the 5% safety threshold, indicating negligible erythrocyte disruption [35]. In contrast, red blood cells in the positive control (Triton X‐100) were completely lysed, producing a red solution, whereas those in the HAPP and HAPPF groups remained intact and evenly dispersed after centrifugation, confirming their excellent blood compatibility.

Calcein‐AM/PI double staining further demonstrated high cell viability of HUVECs cultured in the hydrogel extracts over 24–72 h, with no observable cytotoxicity (Figure 3B and Figure S3). Under oxidative stress induced by Rosup, cells in the HAPPF group exhibited notably improved viability, suggesting that the released H_2_S effectively mitigated ROS‐mediated cellular damage. This cytoprotective effect is attributed to the antioxidant and signaling roles of H_2_S, which may involve modulation of pathways such as NF‐κB, thereby suppressing ROS accumulation and maintaining redox homeostasis.

The wound‐healing assay revealed that after 24 h, the scratch closure rate in the HAPPF group reached 62.82%, markedly higher than that of the HAPP (41.60%), model (21.32%), and control (25.94%) groups (Figure 3C,D). These results indicate that ROS‐triggered H_2_S release from HAPPF significantly promotes HUVEC migration. Meanwhile, the HA backbone may synergistically enhance cell adhesion and motility via CD44‐mediated interactions, creating a favorable microenvironment for H_2_S bioactivityThe Transwell assay further confirmed that HAPPF extracts significantly enhanced the vertical migration of HUVECs compared with the HAPP and control groups (Figure 3E,F and Figure S4). Crystal violet staining and quantitative analysis consistently showed greater cell translocation in the HAPPF group, implying that H_2_S promotes chemotaxis and transmigration, likely through activation of the PI3K/Akt and VEGF signaling pathways [36, 37].

Angiogenesis was evaluated using a tube formation assay (Figure 3G and Figure S5). HUVECs treated with HAPPF hydrogel formed more extensive and interconnected vascular‐like networks than those in the HAPP and control groups. Quantitative analysis (Figure 3H and Figure S6) revealed significantly higher vessel length, node number, and network area in the HAPPF group, confirming that H_2_S release effectively enhances angiogenic activity.

### 2.5 In Vitro Anti‐Inflammatory, Antioxidant, and H_2_S‐Releasing Properties of HAPPF Hydrogels

**FIGURE 4:**
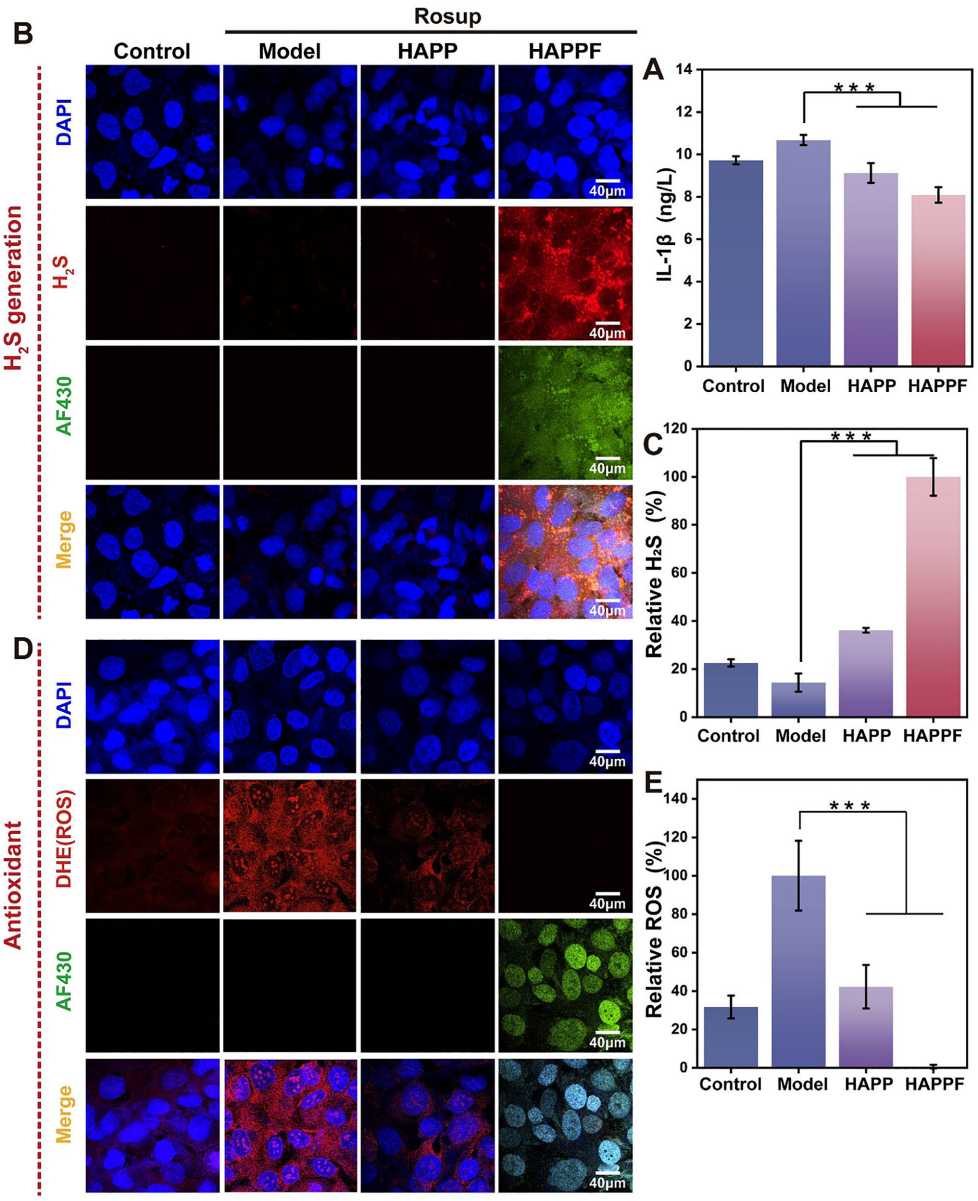
In vitro anti‐inflammatory, antioxidant, and H_2_S‐releasing properties of HAPPF hydrogel. (A) ELISA measurement of IL‐1β expression in HUVECs under different treatments. (B) Representative fluorescence images showing ROS‐stimulated autofluorescence of HSDF‐ NH_2_ and H_2_S‐specific fluorescence detected by Cy‐NO_2_ probe (63 ×, scale bar: 40 µm). (C) Semi‐quantitative analysis of H_2_S fluorescence intensity. (D) Representative images of intracellular ROS levels in HUVECs for different groups (63 ×, scale bar: 40 µm). (E) Semi‐quantitative analysis of ROS fluorescence intensity (*n* = 3 independent samples, ^*^ *p* < 0.05, ^**^ *p* < 0.01, ^***^ *p* < 0.001 by one‐way ANOVA followed by Tukey's post hoc test).

To comprehensively evaluate the in vitro anti‐inflammatory, antioxidant, and H_2_S‐releasing properties of the HAPPF hydrogel, the secretion levels of pro‐inflammatory cytokines TNF‐α, IL‐6, and IL‐1β in HUVECs under different stimuli were first measured. Upon Rosup stimulation, all three cytokines increased by over 10%, indicating oxidative stress‐induced inflammation. In contrast, both HAPP and HAPPF significantly reduced cytokine secretion, with HAPPF showing stronger anti‐inflammatory efficacy, decreasing TNF‐α, IL‐6, and IL‐1β levels by approximately 30.00%, 27.39%, and 24.50%, respectively, compared to 17.10%, 20.18%, and 14.50% in the HAPP group (Figures 3I,J and 4A). These findings indicate that high‐molecular‐weight HA exerts inherent anti‐inflammatory activity, while the ROS‐responsive release of H_2_S from HSDF‐NH_2_ in HAPPF further amplifies this effect. As an endogenous gasotransmitter, H_2_S can suppress the NF‐κB pathway and mitigate oxidative stress, thereby attenuating inflammatory responses.

Subsequently, the intrinsic fluorescence and H_2_S‐releasing behavior of the HAPPF hydrogel were systematically investigated. HSDF‐NH_2_ was activated and emitted a distinct green autofluorescence signal (AF430) under different magnifications (20 × and 63 ×). Following the addition of the H_2_S‐specific probe Cy‐NO_2_, strong red fluorescence was observed in the HAPPF group under high magnification, accompanied by the characteristic AF430 signal from activated HSDF‐NH_2_ (Figure 4B and Figure S7), confirming successful H_2_S generation. Semi‐quantitative analysis of the H_2_S fluorescence channel under high magnification (Figure 4C) revealed significantly higher intensity in the HAPPF group compared with HAPP, indicating that HSDF‐NH_2_ exhibits both robust autofluorescence and highly efficient H_2_S‐releasing capability.

ROS fluorescence imaging further revealed that the model group exhibited the highest ROS signal, whereas HAPPF treatment markedly reduced ROS fluorescence to nearly normal levels (Figure 4D,E and Figure S8). Quantitative data showed approximately 50% ROS reduction in HAPP and near‐complete ROS elimination in HAPPF. This superior antioxidant effect arises from dual mechanisms: ROS consumption by boronate ester bonds in HAPP and further ROS scavenging through ROS‐triggered H_2_S release from HSDF‐NH_2_. Collectively, the HAPPF hydrogel integrates anti‐inflammatory, antioxidant, and self‐reporting fluorescence capabilities via complementary chemical and gaseous mechanisms, offering a promising approach for dynamic regulation of chronic wound microenvironments.

### 2.6 In Vitro Validation of HAPPF Hydrogel Regulating Immune–Vascular–Neural Regeneration and Anti‐Apoptotic Activity

To evaluate the regulatory effects of HAPPF hydrogels on the immune‐vascular‐nerve axis and cell apoptosis, we performed immunofluorescence (IF) staining, ELISA quantification, and flow cytometry. IF imaging revealed that Rosup‐induced oxidative stress severely quenched the fluorescence signals of VEGF, NGF, CGRP, and TGF‐β in HUVECs. This suppression was further corroborated by ELISA data, which showed that the expression levels of VEGF (216.92 ± 51.16 pg/mL), NGF (72.11 ± 5.54 pg/mL), CGRP (8.97 ± 0.15 pg/mL), and TGF‐β (61.61 ± 17.39 pg/mL) dropped to extreme lows, while the apoptosis rate significantly surged to 18.31 ± 2.44%.

**FIGURE 5:**
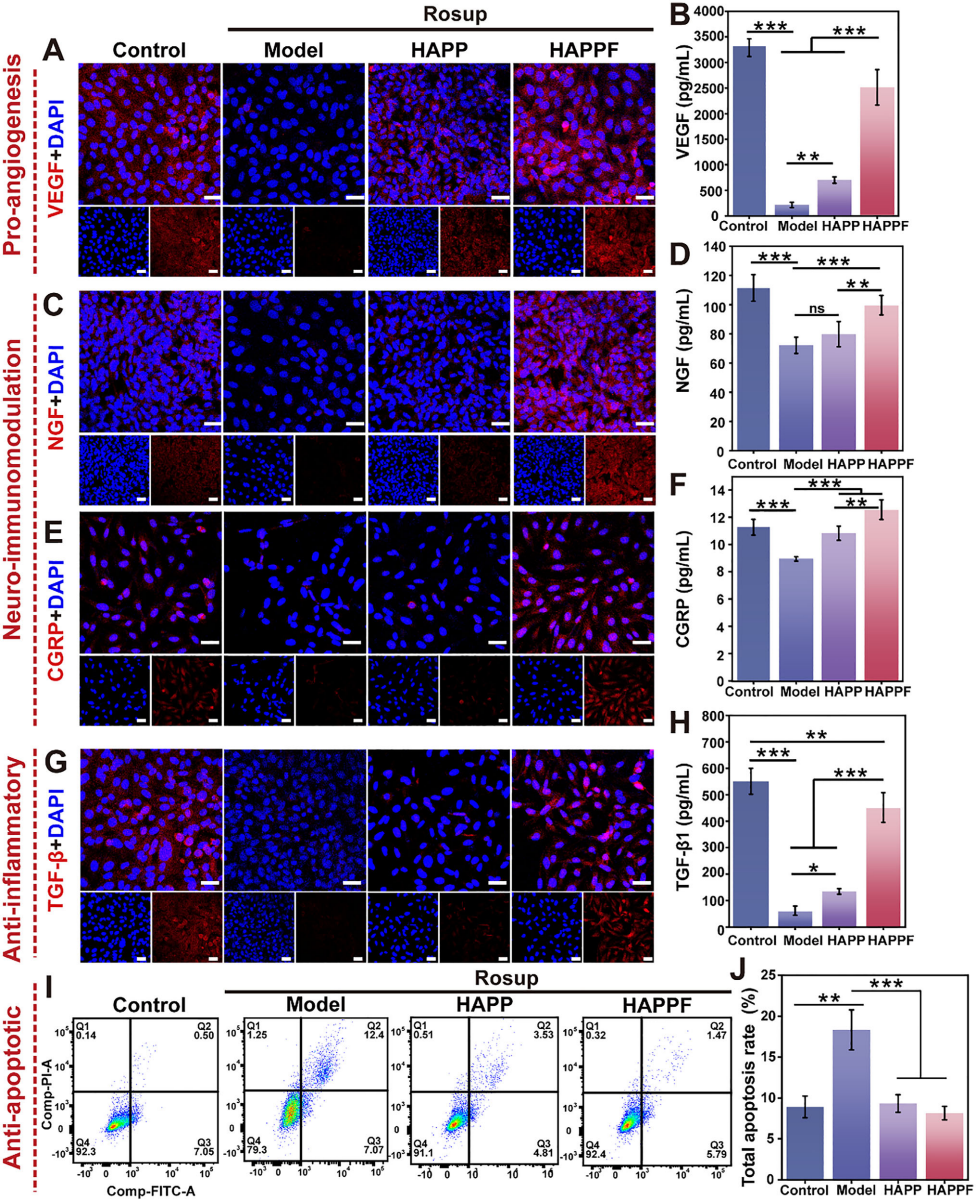
In vitro regenerative and anti‐apoptotic effects of HAPPF hydrogels on HUVECs. (A,C,E,G) Representative immunofluorescence images showing the expression of VEGF (A), NGF (C), CGRP (E), and TGF‐β (G) in HUVECs under different treatments (20 ×, scale bar: 20 µm). (B,D,F,H) ELISA quantification of VEGF (B), NGF (D), CGRP (F), and TGF‐β (H) secretion levels in HUVEC culture supernatants (*n* = 6 independent samples). (I,J) Flow cytometry analysis (I) and corresponding statistical total apoptosis rates (J) of HUVECs under different treatment groups (*n* = 3 independent samples, ^*^ *p* < 0.05, ^**^ *p* < 0.01, ^***^ *p* < 0.001, ns = no significance by one‐way ANOVA followed by Tukey's post hoc test).

Although the HAPP group showed partial recovery—elevating VEGF, NGF, CGRP, and TGF‐β levels to 701.33 ± 62.63 pg/mL, 79.81 ± 8.65 pg/mL, 10.86 ± 0.52 pg/mL, and 134.26 ± 10.48 pg/mL, respectively, and reducing apoptosis to 9.32% ± 1.06%—the most profound therapeutic impact was observed in the HAPPF group. Notably, HAPPF treatment markedly intensified the red fluorescence intensity of these proteins, achieving a more widespread and uniform intracellular distribution compared to both the Model and HAPP groups. Statistical analysis confirmed that these regenerative markers nearly returned to physiological levels: VEGF (2514.68 ± 346.45 pg/mL), NGF (99.61 ± 6.65 pg/mL), CGRP (12.58 ± 0.72 pg/mL), and TGF‐β (451.76 ± 56.04 pg/mL). Concurrently, the apoptosis rate was further suppressed to 8.15% ± 0.82%. These findings underscore the synergistic potential of the HA skeleton and ROS‐responsive H_2_S release in promoting tissue regeneration and inhibiting programmed cell death (Figure 5A–J and Figure S13).

Mechanistically, H_2_S released from HSDF‐NH_2_ within HAPPF hydrogel, as a key endogenous gas signal molecule, cooperatively regulates immune, vascular and neural responses under oxidative stress and inflammatory conditions. We hypothesize that H_2_S can activate the PI3K/Akt/eNOS signaling pathway, enhance VEGF‐mediated angiogenesis, and maintain endothelial cell activity; simultaneously upregulating the expression of CGRP and NGF by regulating MAPK and PPAR signaling [16, 38, 39, 40], thereby maintaining neurovascular coupling and promoting neuroprotection and functional regeneration. In addition, the upregulation of TGF‐β induced by H_2_S suggests its potential role in anti‐inflammatory regulation and extracellular matrix remodeling, which may be related to the inhibition of IL‐17 and NF‐κB signaling, thereby alleviating the inflammatory cascade. The synergistic activation of the above signals not only reconstructed the steady‐state balance of the neuro‐vascular axis, but also significantly reduced the apoptosis rate (Q2 + Q3 quadrant) and promoted cell survival. Overall, H_2_S serves as a signaling hub, integrating transcription pathways such as PPAR, IL‐17, NF—κB, and MAPK to achieve multiple synergistic effects of angiogenesis, nerve regeneration, inflammation relief, and anti‐apoptosis, providing a systematic immune vascular neural regulatory mechanism basis for chronic wound repair [41, 42].

### 2.7 Fluorescence Imaging Performance of HAPPF Hydrogel in Vivo

**FIGURE 6:**
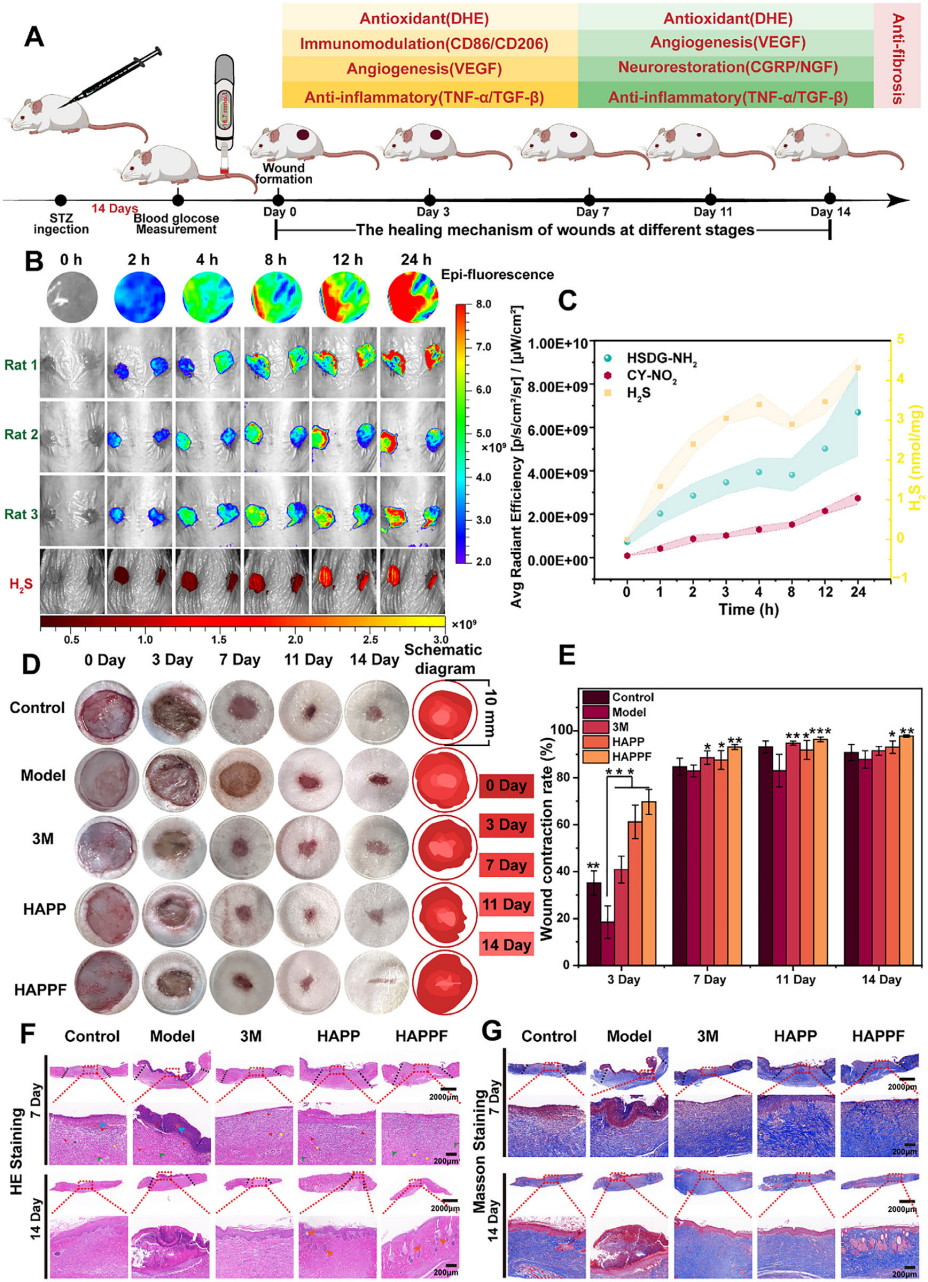
In vivo evaluation of the HAPPF hydrogel in diabetic rats. (A) Schematic illustration of the experimental timeline for the wound healing study.(B) Representative in vivo fluorescence images showing the ROS‐triggered release of HSDG‐ NH_2_ (green) and the response of the H_2_S‐specific probe CY‐NO_2_ (red) at the wound site over 24 h (*n* = 3 independent samples).(C) Quantitative analysis of fluorescence intensities for HSDG‐NH_2_ and CY‐NO_2_, integrated with the actual H_2_S concentration measured in wound skin homogenates (*n* = 6 independent samples).(D) Representative photographs showing the wound healing process under different treatments from day 0 to day 14 (Scale bar: 1 cm).(E) Wound contraction rates measured on days 3, 7, 11, and 14 (*n* = 6 independent samples).(F) Representative H&E‐stained images of wound tissues collected on days 7 and 14. (Black boxes indicate granulation tissue; blue arrows, erythrocytes and hemorrhagic regions; red arrows, eosinophils and neutrophils; yellow stars, newly formed capillaries; green arrows, newly deposited collagen fibers; orange arrows, skin appendages including sebaceous glands and hair follicles.) (G) Representative Masson's trichrome staining images of wound tissues collected on days 7 and 14 (^*^ *p* < 0.05, ^**^ *p* < 0.01, ^***^ *p* < 0.001 by one‐way ANOVA followed by Tukey's post hoc test).

To visualize H_2_S release in vivo, the HAPPF hydrogel was applied to the wound surface of diabetic rats, and temporal fluorescence changes were monitored using an in vivo imaging system (Figure 6B). At 0 h, minimal fluorescence was detected, confirming that the ROS‐triggered transformation of the donor was yet to occur at the wound site. As time progressed, the fluorescence signal increased markedly. Notably, CY‐NO_2_ was employed as a specific fluorescent probe to respond to the generated H_2_S.Quantitative analysis of the imaging data (Figure 6C) showed a gradual rise in average radiant efficiency for both the donor HSDF‐NH_2_ and the released H_2_S (detected via CY‐NO_2_), which reached approximately 6.69 × 10^9^ and 2.74 × 10^9^ [p/s/cm^2^/sr]/[µW/cm^2^] respectively at 24 h. This temporal release was further verified at the histological level; fluorescence staining of skin tissue sections (Figure S14) exhibited a progressive intensification and deep penetration of both green (donor) and red (released H_2_S) signals into the wound bed over 48 h. Crucially, the concentration of H_2_S in the wound skin homogenates was also measured (Figure 6C, yellow curve), revealing a consistent upward trend that peaked at 4.32 nmol/mg at 24 h. This multi‐modal evidence demonstrates that the HAPPF hydrogel enables real‐time visualization of H_2_S production, which is highly correlated with the actual tissue levels.

### 2.8 Wound Healing Performance of HAPPF Hydrocolloid in Diabetes Rats

To systematically evaluate the in vivo wound‐healing efficacy of HAPP and HAPPF hydrogels (Figure 6A), a 10‐mm full‐thickness excisional wound model was established on the dorsal skin of diabetic SD rats. The animals were randomly divided into five groups: PBS (Model), commercial 3 M dressing (positive control), HAPP, HAPPF, and normal rats with skin injury (Control). The hydrogels were applied directly to the wound surface and secured with 3 M dressing. Wound area reduction and histological progression were recorded on days 3, 7, 11, and 14 post‐surgery (Figure 6A).

Over time, progressive wound contraction was observed in all groups. On day 3, the Control group exhibited evident scabbing, while the Model group showed delayed closure with a dry wound bed(Figure 6D). In contrast, HAPP hydrogel maintained an optimal moist microenvironment, facilitating wound contraction through the intrinsic hydration and cell migration‐promoting properties of high‐molecular‐weight HA. The HAPPF group displayed the most remarkable healing response, achieving a wound area reduction rate of 69.73% ± 5.32%, approximately 3.78‐fold higher than the Model group (18.43% ± 2.12%) and 1.7‐fold higher than the 3 M group (40.7% ± 3.5%). These data demonstrate that ROS‐responsive H_2_S release further enhanced the bioactivity of the HA matrix, accelerating wound tissue repair.Quantitative analysis across time points confirmed that the 3 M, HAPP, and HAPPF groups exhibited significantly faster healing than the Control and Model groups. By day 14, wound area reduction rates reached 90.73% ± 3.48% (Control), 87.79% ± 3.71% (Model), 91.50% ± 1.90% (3 M), 97.02% ± 0.75% (HAPP), and 97.75% ± 0.48% (HAPPF) (Figure 6E). Nearly complete re‐epithelialization and enhanced dermal tissue regeneration were observed in the HAPPF‐treated wounds, outperforming the commercial 3 M dressing.

To further systematically evaluate the wound healing process, we performed histological analyses using Hematoxylin and Eosin (H&E) and Masson's Trichrome (Masson) staining on wound tissues from each group. On day 7 post‐injury, both the Control and Model groups exhibited substantial retention of blood cells (blue arrows), suggesting impaired healing progression, which was particularly pronounced in the Model group. Moreover, the Model group showed extensive infiltration of eosinophils and neutrophils (red arrows), indicating a persistent inflammatory response. In contrast, the 3 M, HAPP, and HAPPF groups displayed significantly reduced granulation tissue area (black dashed regions), accompanied by notable capillary formation (yellow triangles) and well‐aligned collagen fiber deposition (green arrows), implying an accelerated repair process (Figure 6F). Quantitative analysis of Collagen Volume Fraction (CVF) at day 7 further confirmed this trend, with the HAPPF group reaching approximately 47.99%, significantly higher than the Model group (*p* < 0.001) and the 3 M group (*p* < 0.05) (Figure S16).

By day 14, the HAPPF group achieved nearly complete re‐epithelialization and exhibited mature skin appendage structures, including sebaceous glands and hair follicles (orange arrows), with tissue architecture closely resembling that of normal skin. Statistically, the re‐epithelialization rate of the HAPPF group reached nearly 100%, exhibiting a marked advantage over the Model group and other treatment groups (*p* < 0.0001) (Figure S15). In contrast, the Model group still showed abundant inflammatory cell infiltration and markedly delayed healing. Collagen metabolism, which plays a critical role throughout the wound repair process, is essential for the restoration of tissue mechanical strength. As illustrated in Figure 6G, collagen deposition on days 7 and 14 further corroborated the histological observations: the Model group still presented residual scab formation, indicating severely delayed diabetic wound healing, whereas the HAPPF group exhibited a clearly defined, intensely red‐stained epidermal layer, significantly enhanced collagen deposition, and more orderly arranged neo‐collagen fibers. The CVF of the HAPPF group remained the highest among all groups at day 14, providing robust support for its superior ability in tissue remodeling (Figure S16). These findings collectively demonstrate that the HAPPF hydrogel effectively modulates the inflammatory phase, promotes angiogenesis, and facilitates collagen remodeling, thereby significantly accelerating the healing of diabetic wounds.

**FIGURE 7:**
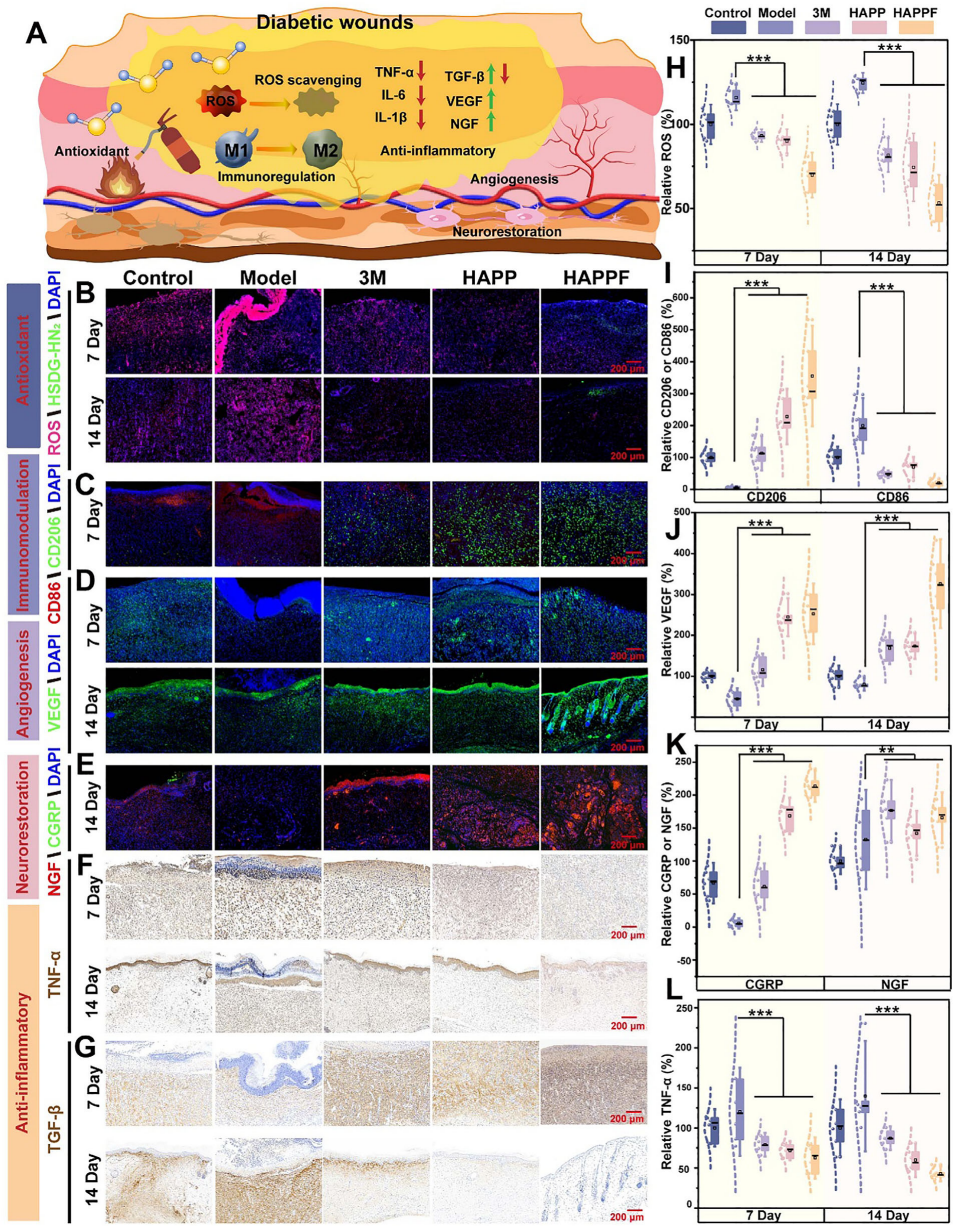
Systematic histopathological and immunofluorescence analysis of wound healing in vivo. (A) Schematic diagram illustrating the H_2_S‐coordinated hierarchical healing cascade involving antioxidant defense, immune rebalancing, angiogenesis, neuroregeneration, and scar suppression in diabetic wound therapy. (B) Representative immunofluorescence images of ROS (red) in wound tissues at days 7 and 14, with green fluorescence indicating HSDF‐ NH_2_ response(Scale bar: 200 µm). (C) Immunofluorescence co‐staining of CD86 (red) and CD206 (green) in wound sections on day 14(Scale bar: 200 µm). (D) Immunofluorescence staining of VEGF in skin tissues at days 7 and 14(Scale bar: 200 µm). (E) Immunofluorescence staining of CGRP (green) and NGF (red) in wound tissues on day 14(Scale bar: 200 µm). (F) Immunohistochemical staining of TNF‐α in skin tissues on day 14(Scale bar: 200 µm). (G) Immunohistochemical staining of TGF‐β in skin tissues on day 14(Scale bar: 200 µm). (H–L) Quantitative analysis of the relative expression levels of ROS (H), CD86 and CD206 (I), VEGF (J), CGRP and NGF (K), and TNF‐α (L) at the corresponding time points (*n* = 6 independent samples, ^*^ *p* < 0.05, ^**^ *p* < 0.01, ^***^ *P* < 0.001 by one‐way ANOVA followed by Tukey's post hoc test).

To further elucidate the temporospatial regulatory mechanism of HAPP and HAPPF hydrogels in diabetic wound repair, we performed a systematic analysis of wound tissues on postoperative days 7 and 14. ROS staining revealed that both hydrogels effectively alleviated oxidative stress within the wound microenvironment, with HAPPF exhibiting the most pronounced antioxidant activity, accompanied by visible green fluorescence of HSDG‐NH_2_. By day 14, the ROS level in the HAPPF group decreased by approximately 30% compared with the model group (*p* < 0.001) and by 21.19% relative to the HAPP group (Figure 7B,H and Figure S17). The attenuation of oxidative stress provided a favorable foundation for immune homeostasis remodeling. Immunofluorescence further demonstrated that HAPPF markedly suppressed pro‐inflammatory M1 macrophages (CD86; reduced to 10% of model, *p* < 0.001) while promoting anti‐inflammatory M2 polarization (CD206; increased 62.5‐fold vs. model, *p* < 0.001), resulting in a significant elevation of the M2/M1 ratio (Figure 7C,I and Figure S18).

Building on immune modulation, HAPPF significantly enhanced angiogenesis and tissue regeneration. VEGF expression in HAPPF‐treated wounds was elevated 2.52‐fold on day 7 and further increased to 3.26‐fold on day 14 compared with the model (*p* < 0.001) (Figure 7D,J and Figure S19). ELISA results confirmed this trend, showing that VEGF concentration in the HAPPF group reached 132.11 pg/mL by day 14, representing a 1.92‐fold increase relative to the model (68.65 pg/mL) (*p* < 0.001) (Figure S20). Concurrently, neural regeneration markers CGRP and NGF were upregulated 42.01‐fold and 1.25‐fold in fluorescence intensity, respectively. Quantitative ELISA data further supported this neurorestorative effect: CGRP and NGF levels in the HAPPF group were elevated to 42.90 pg/mL (1.78‐fold vs. model) and 65.02 pg/mL (1.81‐fold vs. model), respectively (*p* < 0.001) (Figure 7E,K and Figures S21 and S22). These findings align with the recently proposed “neuro‐immune‐vascular axis” model, wherein H_2_S acts as a pivotal mediator bridging neural and vascular regeneration under precise immune regulation.

Furthermore, ELISA and immunohistochemistry of TNF‐α and TGF‐β indicated that HAPPF possesses temporally regulated anti‐inflammatory and antifibrotic properties (Figure 7F,G,L and Figures S23–S25). On day 7, TGF‐β expression in HAPPF‐treated wounds was 2.82‐fold higher than the model, promoting extracellular matrix deposition and epithelialization. Specifically, ELISA quantification showed TGF‐β levels in the HAPPF group reached 124.42 pg/mL on day 7 (2.23‐fold vs. model). By day 14, TGF‐β levels returned near baseline, while TNF‐α concentration significantly dropped to 31.78 ng/L (a 70.56% reduction vs. model). This demonstrates a biphasic regulatory feature of HAPPF, activating regenerative signals in the early phase while suppressing fibrosis in the later stage.

Collectively, HAPPF hydrogel orchestrates a hierarchical and coordinated healing cascade through ROS‐responsive H_2_S release, encompassing antioxidant defense, immune homeostasis, angiogenesis, neuroregeneration, and scar inhibition. By activating a systemic modulating of the immuno–vascular–neural axis, H_2_S serves as a central bioactive mediator that accelerates wound closure, promotes functional tissue reconstruction, and mitigates scar formation.

The in vivo biocompatibility and degradation profiles of the hydrogels were systematically evaluated. Following subcutaneous implantation, the HAPPF hydrogel exhibited a progressive and controlled biodegradation, with the residual weight percentage gradually decreasing over the study period (Figure S26). Histological analysis of the tissues surrounding the implants revealed no significant chronic inflammatory infiltration or severe fibrous encapsulation at the interface between the hydrogel and host tissue (Figure S27). This progressive integration with the surrounding tissue, coupled with the consistent degradation kinetics, underscores the excellent localized biocompatibility of the HAPPF system. Furthermore, systemic toxicity was assessed through hematological and major organ toxicity evaluations. Blood analysis revealed no significant differences among HAPP‐, HAPPF‐treated, and healthy control groups across 21 parameters, including white blood cell count, red blood cell count, hemoglobin concentration, and platelet count (Figure S28), indicating an absence of systemic toxicity. Furthermore, H&E staining of major organs—including heart, liver, spleen, lung, and kidney—showed normal histological architecture without observable alterations compared with healthy controls (Figure S29), confirming that hydrogel treatment did not induce organ toxicity. Collectively, these results demonstrate excellent in vivo biocompatibility, safety, and desirable degradability of HAPP and HAPPF hydrogels, providing robust preclinical evidence supporting their potential for chronic wound applications. The current study is nevertheless limited by the absence of long‐term follow‐up, repeated dosing evaluation, and comprehensive assessment of sustained therapeutic exposure. These limitations have been explicitly acknowledged in the revised Discussion section. Future studies will further systematically investigate the long‐term therapeutic stability, optimal administration regimen, and comprehensive biosafety of this delivery system to support its potential clinical translation.

**FIGURE 8:**
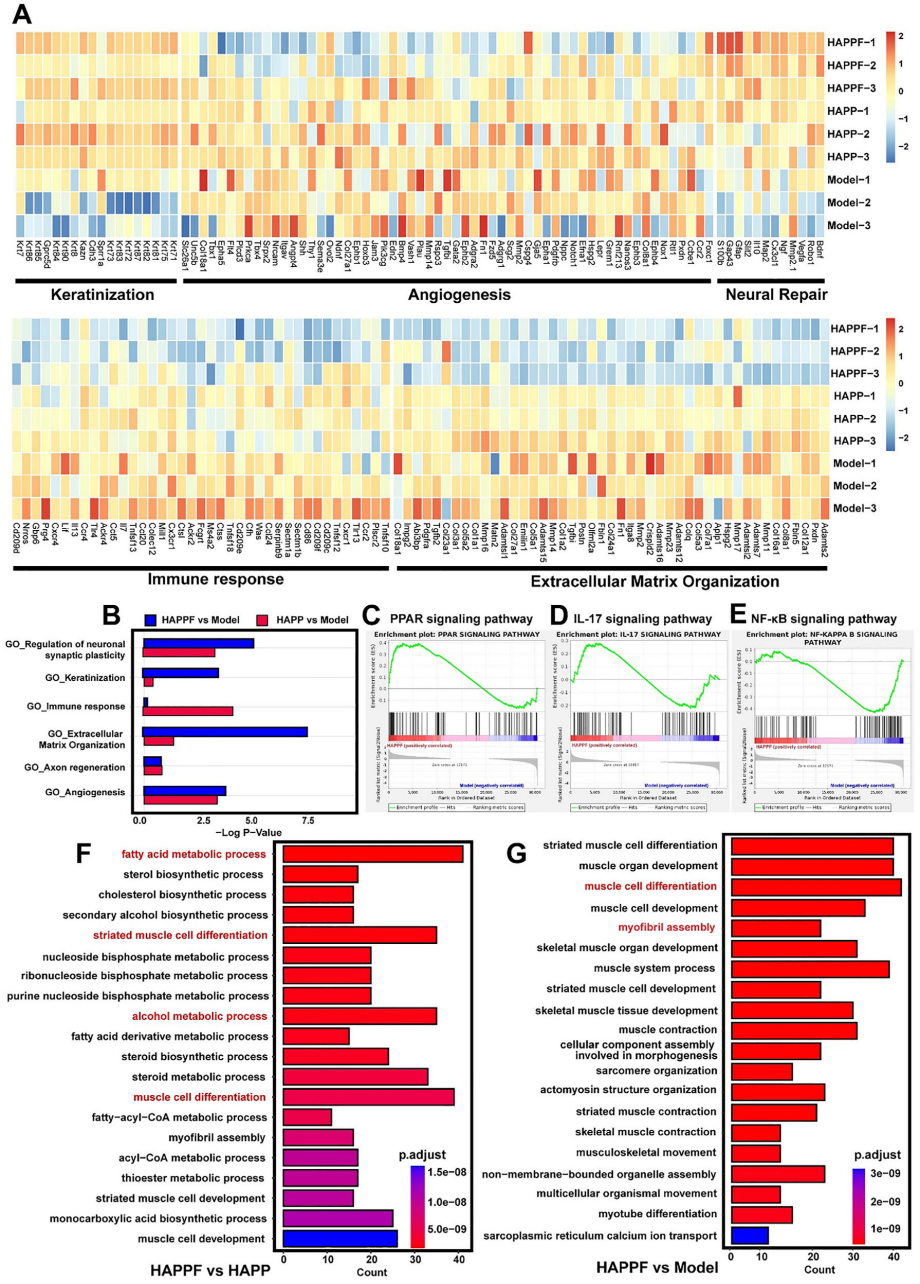
Transcriptomic analysis (bulk RNA sequencing) of wound tissues.(A) Heatmap of differentially expressed genes related to wound healing processes on day 14, including keratinization, angiogenesis, neuroregeneration, immune response, and ECM remodeling.(B) GOenrichment analysis of biological processes in HAPP and HAPPF hydrogel‐treated groups compared with the model group. HAPPF treatment notably upregulated gene sets associated with neuronal regulation, keratinization, angiogenesis, and ECM reconstruction.(C–E) GSEA showing significant modulation of PPAR, IL‐17, and NF‐κB signaling pathways by HAPPF treatment.(F) Enriched biological processes in HAPPF hydrogel‐treated group compared with HAPP hydrogel‐treated group.(G) Enriched biological processes in HAPPF hydrogel‐treated group compared with Model group(*n* = 3 independent samples).

To elucidate the molecular mechanisms by which HAPP and HAPPF hydrogels promote diabetic wound healing and suppress scar formation, bulk RNA sequencing (RNA‐seq) was performed on wound tissues collected at day 14 (late repair phase). Principal component analysis (PCA) initially revealed distinct clustering patterns, indicating systematic transcriptomic shifts across groups (Figure 8A). A comprehensive classification of differentially expressed genes (DEGs) further demonstrated that HAPPF treatment induced multi‐dimensional biological modulation, particularly in keratinization, angiogenesis, neural regeneration, and immunomodulation. Volcano plots (|log_2_FC| > 1, adjusted *p* < 0.05) quantified this impact: compared to the Model group, HAPPF treatment identified 538 DEGs (304 upregulated and 234 downregulated; Figure S30B). Notably, a direct comparison between the two hydrogel groups identified 181 genes significantly upregulated in HAPPF relative to HAPP (Figure S30D). These specific DEGs, including functional markers such as Mybpc2, Pygm, and Adipoq, highlight the unique biological “boost” provided by sustained H_2_S release in promoting functional tissue reconstruction. Heatmap and Gene Ontology (GO) enrichment analyses further confirmed that HAPPF robustly enhanced processes related to neuronal regulation, keratinization, and extracellular matrix (ECM) organization (Figure 8B). In contrast, HAPP primarily modulated immune‐related pathways, likely due to its intrinsic scaffold properties. These findings collectively demonstrate that HAPPF synergistically facilitates immunomodulation, neovascularization, and neural regeneration.

Gene Ontology (GO) and GSEA enrichment analyses identified the PPAR, IL‐17, NF‐κB, and TGF‐β pathways as central molecular hubs (Figure 8B–E and Figures S31 and S32). Notably, KEGG enrichment analysis (Figure 8F,G) further revealed that HAPPF predominantly influenced fatty acid metabolism, general metabolic pathways, and steroid biosynthesis, providing new insights into H_2_S ‐mediated metabolic modulating. HAPPF showed a much more pronounced enrichment in the PPAR signaling pathway compared to HAPP, suggesting that H_2_S acts as a potent metabolic switch. This was quantitatively confirmed by day‐14 ELISA: HAPPF increased PPARγ levels to 311.81 ±12.55 pg/mL, a 1.37‐fold increase over the Model (*p* < 0.05 vs. HAPP) (Figure S33A). Integrating these findings, we propose that H_2_S orchestrates healing through an “immune–vascular–neural axis.” At the oxidative stress level, H_2_S suppresses ROS‐sensitive NF‐κB and MAPK activation to prevent chronic inflammation. Simultaneously, H_2_S modulates lipid metabolism and mitigates excessive inflammation via PPARγ activation, consequently fine‐tuning TGF‐β signaling to avoid excessive collagen deposition and scar formation [43, 44].

The anti‐inflammatory superiority of HAPPF was evidenced by a 4.42‐fold reduction in IL‐17A (10.67 ± 4.69 pg/mL) and a 2.7‐fold reduction in iNOS compared to the Model, significantly outperforming the HAPP group (Figure S33B,D). Most importantly, the neuro‐immune interaction was reinforced by the dramatic 2.51‐fold upregulation of BDNF in the HAPPF group (39.55 ± 7.96 pg/mL) compared to HAPP (Figure S33C). Collectively, our analysis identifies TGF‐β signaling as a central molecular hub through which H_2_S modulates the PPARγ/NF‐κB/BDNF axis, reprograms diabetic wound healing, and suppresses fibrotic scar formation [42, 45].

### 2.9 HAPPF Hydrogel Alleviates Hypertrophic Scarring in a Diabetic Rabbit Ear Model via Anti‐Fibrotic Signaling Regulation

**FIGURE 9:**
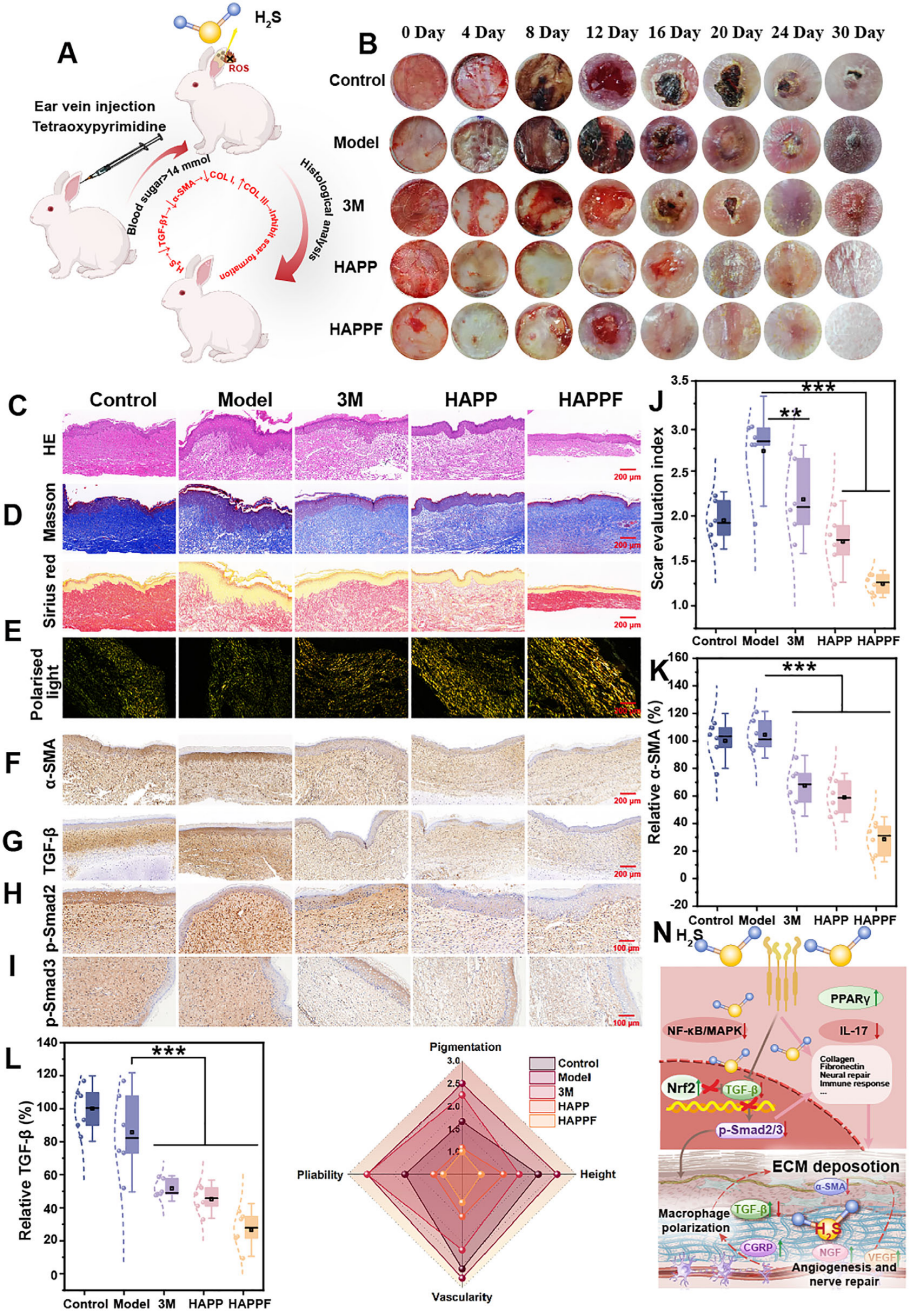
In vivo evaluation of HAPP and HAPPF hydrogels in a diabetic rabbit ear hypertrophic scar (HS) model. (A) Schematic illustration of the H_2_S‐mediated therapeutic process for HS repair in the diabetic rabbit ear model. (B) Representative photographs of wound healing and scar formation at different time points (0–30 days). (C–E) Representative images of scar tissues from different treatment groups stained with (C) H&E, (D) Masson's trichrome, and (E) Sirius Red (observed under polarized light) to evaluate collagen organization and alignment (scale bars: 200 µm). (F–I) IHC staining of key markers in scar tissues at day 30, including (F) α‐SMA, (G) TGF‐β, (H) p‐Smad2, and (I) p‐Smad3 (scale bars: 200 µm for F‐G, 100 µm for H‐I). (J) Quantitative analysis of the SEI. (K–L) Quantification of (K) α‐SMA and (L) TGF‐β expression levels in scar tissues (*n* = 6 independent samples, ^*^ *p* < 0.05, ^**^ *p* < 0.01, ^***^ *p* < 0.001 by one‐way ANOVA followed by Tukey's post hoc test.). (M) Vancouver Scar Scale (VSS) radar chart comparing pigmentation, pliability, vascularity, and scar height among different groups. (N) Schematic illustration of the H_2_S‐centered “neuro–immune–vascular” regulatory mechanism underlying scar suppression through the inhibition of TGF‐β/Smad and NF‐κB/MAPK signaling pathways.

To further evaluate the therapeutic efficacy of HAPP and HAPPF hydrogels in improving wound healing and reducing scar formation, a diabetic rabbit ear wound model was established. Due to the absence of significant wound contraction and relatively delayed re‐epithelialization, this model is widely used to partially recapitulate key features of human hypertrophic scar formation. Diabetes was induced by auricular vein injection of alloxan (blood glucose > 14 mmol/L for seven consecutive days), followed by the creation of full‐thickness circular skin defects (15 mm in diameter) on the dorsal ear surface to establish reproducible scar‐prone wounds (Figure 9A).

HAPPF treatment markedly accelerated wound closure and effectively prevented excessive scar formation. By day 16, wounds treated with HAPPF were nearly fully epithelialized, while those in the model group remained partially open. At day 30, dark red, firm, and elevated hypertrophic scars were observed in the model group, whereas HAPPF‐treated wounds exhibited smooth, uniformly pigmented skin with elasticity comparable to the surrounding tissue(Figure 9B). Quantitative analysis revealed that the average scar area in the control group was approximately 314 mm^2^ (≈50% of the original wound area), whereas that in the HAPPF group was reduced by ∼73% (*p* < 0.001).

Scar quality was further assessed using the VSS, evaluating pigmentation, pliability, vascularity, and height. The HAPPF group achieved a mean score of 0.95 ± 0.08, significantly lower than those of the model (2.50 ± 0.36) and HAPP (1.22 ± 0.25) groups (Figure 9M). Histological examination revealed that scars in the HAPPF group were flatter with more parallel and organized collagen fibers (Figure 9C,D and Figure S34). Sirius Red staining was employed to differentiate collagen type I (COL I, red) and type III (COL III, green) within the regenerated tissue. In the model group, a limited amount of green fibers (COL III) with sparse red fibers (COL I) indicated incomplete matrix reconstruction and delayed maturation. In contrast, the HAPPF‐treated wounds exhibited abundant and well‐distributed red and green fibers, reflecting a coordinated increase in both COL I and COL III (Figure 9E and Figure S35). This pattern suggested that HAPPF not only promoted early regenerative COL III deposition but also facilitated the orderly remodeling of COL I, leading to a more physiological collagen ratio and reduced fibrosis. Quantitatively, the scar elevation index (SEI) decreased by approximately 54%, and the total collagen deposition was reduced by ∼58% compared with the model group (Figure 9J). Collectively, these findings demonstrate that HAPPF hydrogel effectively modulated collagen remodeling, enhanced scar maturity, and improved overall scar quality.

To elucidate the underlying molecular mechanisms of tissue remodeling, we performed immunohistochemical staining for key components of the fibrotic signaling cascade (Figure 9F–I). TGF‐β and its downstream mediators, p‐Smad2 and p‐Smad3, are central drivers of fibroblast hyperactivation and pathological scar hypertrophy. Specifically, active TGF‐β triggers the phosphorylation and nuclear translocation of Smad2 and Smad3, which collectively initiate the transcription of profibrotic genes, including α‐SMA. At day 30, expression levels of active TGF‐β and p‐Smad2/3 in the Model group were markedly elevated (100%), corresponding with a significant scar evaluation index and high α‐SMA expression. In contrast, HAPPF treatment demonstrated a profound capacity to modulate this axis. Relative to the Model group, HAPPF reduced active TGF‐β levels by approximately 59.17% and, crucially, led to dramatic decreases in p‐Smad2 (by 71.87%) and p‐Smad3 (by ～76.57%) (Figure 9L and Figure S36). This robust inhibition of Smad phosphorylation effectively suppressed the expression of α‐SMA (by ～75.96%), significantly limiting myofibroblast differentiation (*p* < 0.001 vs. Model) (Figure 9K). These results, combined with transcriptomic data, demonstrate that H_2_S released from the HAPPF hydrogel interrupts the profibrotic feed‐forward loop by blocking the TGF‐β/SMAD2/3 signaling axis. By preventing the excessive nuclear translocation of p‐Smad2/3, HAPPF inhibits fibroblast hyperactivation and the subsequent disordered extracellular matrix deposition (Figure 9G–I). This regulatory mechanism is essential for mitigating late‐stage fibrosis and directing tissue remodeling toward functional regeneration. Collectively, these findings suggest that H_2_S‐mediated coordination of the “immuno–vascular–neural axis” promotes an organized healing process and effectively suppresses excessive scar formation in diabetic wounds(Figure 9N).

## 3 Conclusion

In this study, we developed a ROS‐responsive, H_2_S‐releasing dynamic hydrogel (HAPPF), exhibiting robust mechanical properties and intrinsic self‐healing capability, enabling localized, sustained, and controllable bioactive H_2_S delivery at the wound site. The hydrogel's dynamic network ensures structural resilience, conformal coverage, and adaptability to the hostile and mechanically dynamic chronic wound microenvironments. Mechanistic studies demonstrated that ROS‐activated HSDF‐ NH_2_‐mediated H_2_S release orchestrates the neuro‐immune‐vascular niche by alleviating oxidative stress, modulating macrophage polarization from the pro‐inflammatory M1 phenotype toward the pro‐reparative M2 phenotype, promoting angiogenesis through VEGF upregulation, enhancing neuroregeneration via CGRP and NGF signaling, and exerting spatiotemporally regulated immunomodulation through coordinated modulation of TGF‐β and TNF‐α to suppress fibrosis. Transcriptomic analyses further confirmed the activation of gene programs associated with keratinization, ECM remodeling, and key signaling pathways, including PPAR, IL‐17, and NF‐κB, supporting highly coordinated, sequential tissue repair. Collectively, HAPPF enables multidimensional and hierarchical wound healing by accelerating wound closure, improving tissue remodeling, and mitigating scar formation. These findings highlight the strong translational promise of HAPPF for diabetic chronic wounds therapy and provide a generalizable design paradigm for responsive biomaterials that integrate neuro–immune–vascular regulatory cues. Future studies will focus on fine‐tuning H_2_S release kinetics, exploring synergistic combination strategies with complementary bioactive agents, and systematically evaluating long‐term biosafety and therapeutic efficacy in large‐animal models, thereby facilitating clinical translation.

## 4 Materials and Methods

### 4.1 Materials

Hyaluronic acid (HA, 400–800 kDa) and PVA224 were purchased from Macklin Biochemical Co., Ltd. (Shanghai, China). Phenylboronic acid (PBA), 2‐(N‐morpholino)ethanesulfonic acid (MES), and N‐hydroxysuccinimide (NHS) were obtained from Shanghai Bide Pharmatech Ltd. (Shanghai, China), and 1‐Ethyl‐3‐(3‐dimethylaminopropyl) carbodiimide hydrochloride (EDC·HCl) from Heowns Opde Technology Co., Ltd. (Tianjin, China). Potassium bromide and Triton X‐100 were purchased from Macklin Biochemical Co., Ltd. ELISA kits for VEGF, NGF, brain‐derived neurotrophic factor (BDNF), interleukin‐17A (IL‐17A), and transforming growth factor‐beta (TGF‐β) were supplied by UcallM Life Science & Technology Co., Ltd. (Hangzhou, China). ELISA kits for calcitonin gene‐related peptide (CGRP), inducible nitric oxide synthase (iNOS), and peroxisome proliferator‐activated receptor gamma (PPAR‐γ) were obtained from Elabscience Biotechnology Co., Ltd. (Wuhan, China). The H_2_O_2_ assay kit was supplied by Solarbio Science & Technology Co., Ltd. (Beijing, China). MTT and Calcein‐AM/PI assay kits were obtained from Beyotime Biotechnology (Shanghai, China). Matrigel matrix was purchased from Corning Inc. (NY, USA). Primary antibodies used for immunofluorescence and immunohistochemistry included VEGF (ab32152, Abcam), NGF (CY5217, Abways), and CGRP (BS‐10639R, Bioss), with Alexa Fluor 647‐conjugated goat anti‐rabbit IgG (ab150079, Abcam) as secondary antibody. All other reagents were of analytical grade and used as received. Deionized water was used throughout the study.

### 4.2 Synthesis and Characterization of the HA‐PBA Conjugate

The HA‐PBA conjugate was synthesized via a carbodiimide‐mediated coupling reaction. Briefly, 8 g of hyaluronic acid (HA, 400–800 kDa) was completely dissolved in 400 mL of 0.5 m MES buffer (pH 5.0) under constant stirring at 45°C. Then, 4.6 g of EDC·HCl and 2.8 g of NHS were added to the solution and allowed to dissolve fully to activate the carboxyl groups of HA. This was followed by the addition of 3.2 g of 3‐aminophenylboronic acid (PBA). The reaction was allowed to proceed at 45°C for 24 h under continuous stirring. The resulting mixture was purified by dialysis against deionized water using a dialysis membrane (MWCO: 8 kDa) for 72 h. The final HA‐PBA product was obtained as a white, porous solid after lyophilization for 3 days.

The chemical structure of HA‐PBA was verified by ^1^H‐NMR spectroscopy (Bruker Advance 400, 400 MHz) using deuterium oxide (D_2_O) as the solvent. Spectral data were processed and analyzed using MestReNova software (v14.0.0). Fourier‐transform infrared (FT‐IR) spectroscopy (JASCO FT/IR‐4600) was employed to further characterize the chemical bonds in HA, PBA, HA‐PBA, and HAPP samples. Measurements were performed using the KBr pellet method, with scans collected in the range of 4000–400 cm^−^^1^. Thermal properties were evaluated by differential scanning calorimetry (DSC). Samples (∼5 mg) were sealed in standard aluminum pans and heated from 25°C to 300°C at a rate of 10°C/min under a nitrogen atmosphere.

### 4.3 Preparation of HAPP and HSDF‐NH_2_‐Loaded HAPPF Hydrogels

The HAPP hydrogel was formed via spontaneous cross‐linking between the phenylboronic acid groups of HA‐PBA and the diol groups of PVA. A precursor solution of HA‐PBA (3 wt.%) and PVA solutions (2, 3, and 4 wt.%) were prepared separately in phosphate‐buffered saline (PBS, pH 7.4). The hydrogels were prepared by mixing the HA‐PBA solution with each PVA solution at a 1:1 volume ratio, followed by immediate vortexing. Gelation occurred rapidly, yielding hydrogels designated as HAPP1, HAPP2, and HAPP3, corresponding to the increasing PVA concentration. Based on prior optimization, HAPP2 was further modified with HSDF‐ NH_2_ at a final concentration of 200 µm, and the resulting functionalized hydrogel was named HAPPF.

### 4.4 Morphologies Analysis of Hydrogels

The microporous morphology of the freeze‐dried hydrogels was examined using a scanning electron microscope (SEM, Hitachi SU8010). Prior to imaging, the hydrogel samples were mounted on metallic stubs using conductive carbon tape and sputter‐coated with a thin layer of gold under vacuum to enhance conductivity. The internal pore structure, including pore size and architecture, was systematically imaged and compared for HAPP and HAPPF hydrogels with varying compositions.

### 4.5 Measurement of Swelling Ratio and Moisturizing Properties

To evaluate the swelling behavior, hydrogel samples (50 mg) were immersed in 5 mL of deionized water at 37°C. At predetermined time intervals, the samples were removed, gently blotted to eliminate surface water, and weighed. The swelling ratio was calculated according to Equation (1):

(1) $$\mathrm{The}\;\mathrm{swelling}\;\mathrm{rate}\;\;\left(\%\right)=\frac{W_{0}-W_{t}}{W_{0}}\;\times 100\%$$

where *W_t_* and *W_0​_* represent the weights of the swollen and initial dry hydrogels, respectively.

Moisturizing properties were assessed by placing hydrogel specimens (0.2 g) in open Petri dishes under 30% relative humidity at room temperature. The mass was recorded at selected time intervals, and the water retention was expressed as the residual mass, calculated using Equation (2):

(2) $$Residual\;mass\;\left(\%\right)=\frac{W_{t}}{W_{0}}\;\times \;100\%$$

where *W_t_* represents the residual mass of the hydrogel at each time point, and *W_0​_* is the initial hydrogel mass.

### 4.6 Rheological Characterization of HAPP Hydrogels

Rheological measurements were conducted on a rotational rheometer (HAAKE MARS 60, Thermo Fisher Scientific, China) with a 25 mm parallel‐plate geometry at 37°C. The linear viscoelastic region (LVR) was determined by amplitude sweep, followed by frequency sweeps (0.1–10 Hz) to evaluate storage (G′) and loss (G″) moduli. Self‐healing ability was assessed by step‐strain tests with alternating low (1%, within LVR) and high (1000%, beyond LVR) strain cycles. Creep–recovery, steady shear flow, and yield stress analyses were performed to characterize time‐dependent viscoelasticity, shear‐thinning behavior, and yielding properties, respectively. Thixotropic recovery was further examined by applying low–high–low shear rate cycles to probe reversible microstructural reconstruction.

### 4.7 Optical Microscopy Observation of Self‐Healing Behavior

The self‐healing behavior of hydrogels was directly observed using an upright optical microscope (BX53, Olympus, Japan) at room temperature. Hydrogel specimens were cut into two halves with a sterile scalpel, immediately rejoined, and placed on a glass slide without external force. The healing interface was continuously monitored in bright‐field mode, and images were captured at predefined intervals (0, 5, 10, 20, and 30 s) to track the dynamic reconnection of the fractured surfaces. The healing extent was qualitatively evaluated based on the disappearance of the interface and the recovery of structural continuity.

### 4.8 Controlled Release of HSDF‐NH_2_ From HAPPF Hydrogels Under Simulated Wound Microenvironment

The release behavior of HSDF‐NH_2_ from HAPPF hydrogels was evaluated under conditions simulating various wound microenvironments. Hydrogel samples (1 mL) containing 200 µm HSDF‐NH_2_ were incubated in 40 mL of different release media at 37°C, including: PBS at pH *7.4* and *6.0*; PBS supplemented with 5 mg/mL glucose at pH *7.4* and *6.0*; PBS supplemented with 1 mm H_2_O_2_ at pH *7.4* and *6.0*; and PBS containing both 5 mg/mL glucose and 100 mm H_2_O_2_ at pH *7.4* and *6.0*. At predetermined time intervals, 1 mL of release medium was withdrawn and replaced with an equal volume of fresh medium. The concentration of HSDF‐NH_2_ in the collected aliquots was determined spectrophotometrically at 385 nm. Cumulative release was calculated according to Equation (3):

(3) $$\mathrm{Commulative}\mathrm{release}(\%)=(\mathrm{Cn}\times V1+\sum_{i=1}^{n-1}\mathrm{Ci}\times V2)/m\times 100\%$$

where C_n_: concentration measured at the n sampling point, V_1_: release medium volume, V_i_: concentration measured at the i sampling point, V_2_: Volume of sampling, m: The weight of HSDF‐NH_2_ in the hydrogel.

### 4.9 Real‐Time Monitoring and Quantification of H_2_S Release

To semi‐continuously track the H_2_S release profile of HSDF‐ NH_2_‐loaded hydrogels under oxidative conditions, 500 µL of HAPPF hydrogel was incubated in 5 mL PBS (pH *7.4*) containing 1 mM H_2_O_2_ at 37 °C. Fluorescence intensity of the supernatant was measured at predetermined intervals (Ex = 427 nm) using fluorescence spectroscopy. Parallel experiments were conducted for quantitative H_2_S determination via the methylene blue method: at each time point, 200 µL of supernatant was mixed with 50 µL of 1% zinc acetate and 50 µL of 0.1 m N,N‐dimethyl‐p‐phenylenediamine sulfate in 3 m HCl, followed by addition of 50 µL of 0.1 m FeCl_3_ in 1.2 m HCl. After 30 min incubation at room temperature, absorbance at 670 nm was recorded and H_2_S concentration was determined using a standard curve.

The fluorescence intensity exhibited a strong positive correlation (R^2^ > 0.99) with the chemically determined H_2_S concentrations, validating the fluorescence signal as a reliable semi‐quantitative indicator for real‐time visualization of H_2_S release dynamics from the hydrogel matrix.

### 4.10 H_2_O_2_ Scavenging Performance Test

The H_2_O_2_ scavenging activity of HAPP and HAPPF hydrogels containing varying concentrations of HSDF‐NH_2_ was evaluated using a commercial hydrogen peroxide detection kit. Briefly, 200 µL of hydrogel was incubated with 800 µL of PBS containing 200 µM H_2_O_2_ at 37°C. At predetermined time points, the residual H_2_O_2_ concentration in the supernatant was quantified according to the manufacturer's instructions. This approach allowed comparative assessment of the antioxidant capability of blank HAPP and HSDF‐NH_2_‐loaded HAPPF hydrogels, providing insight into the effect of drug loading on H2O2 scavenging efficiency.

### 4.11 Hemostatic, Adhesive, and Tensile Characterization of HAPPF Hydrogels

The hemostatic performance of HAPPF hydrogels was evaluated using tail and liver bleeding models in SD rats. For tail bleeding, anesthetized rats underwent a standardized tail transection, and 300 µL of hydrogel was applied; hemostasis time and total blood loss were recorded. For liver bleeding, nine male SD rats were randomly assigned to three groups (Control, HAPP, HAPPF). Rats were anesthetized with isoflurane (1.5 L/min), and the liver was exposed via a midline abdominal incision. Surrounding exudates were removed, and pre‐weighed filter paper (W_0_) was placed beneath the liver. A scalpel was used to create an incision, inducing bleeding. Hydrogel samples were applied to cover the incision sites in the HAPP and HAPPF groups, while the control group remained untreated. The wound sites were photographed at 0, 30, and 60 s. After 60 s, the filter paper was weighed (W_t_), and blood loss was calculated using Equation (4):

(4) $$\mathrm{Lost}\;\mathrm{blood}\;\left(\mathrm{mg}\right)=\mathrm{Wt}--W0$$

Macroscopic tensile properties were assessed by manually stretching hydrogel samples between fingers to qualitatively evaluate elasticity. Adhesive performance was tested on freshly excised wet rat tissues (heart, liver, spleen, lung, kidney). Hydrogels were placed onto the moist tissue surfaces, and adhesion was visually documented through photographs to assess performance under physiological‐like wet conditions.

### 4.12 Preparation of Hydrogel Extract

Hydrogels were placed in dialysis bags (molecular weight cut‐off 1000 Da) and immersed in cell culture medium at room temperature. After 48 h, the medium was filtered through a 0.22 µm membrane to remove any residual bacteria. The resulting hydrogel extract was collected, aseptically sealed, and stored at 4°C until further use. This procedure ensured the preparation of sterile, cell‐compatible hydrogel extracts for subsequent biological assays.

### 4.13 Cell Culture

Human umbilical vein endothelial cells (HUVECs, RRID: CVCL_2959) were obtained from the Cell Bank of the Chinese Academy of Sciences (Shanghai, China) and were authenticated by short tandem repeat (STR) profiling. Cells were maintained in DMEM/F‐12 medium (Gibco) supplemented with 10% fetal bovine serum (FBS), 100 U/mL penicillin, and 100 µg/mL streptomycin (Gibco). Cells were incubated at 37°C in a humidified atmosphere containing 5% CO2 and 95% air and subcultured upon reaching 80–90% confluence.

### 4.14 Biocompatibility Evaluation of Hydrogels

The cytocompatibility of HAPPF hydrogels was assessed using both MTT and Live/Dead staining assays. HUVECs were seeded in 96‐well plates at a density of 1 × 10^4^ cells per well and allowed to adhere for 12 h. Cells were then incubated with hydrogel extracts for 24 h. For the MTT assay, 20 µL of MTT solution (5 mg/mL) was added to each well, followed by incubation for 4 h to allow formation of formazan crystals. The crystals were subsequently solubilized in DMSO, and absorbance was measured at 570 nm using a microplate reader. Cell viability was expressed relative to untreated controls to quantitatively evaluate cytocompatibility.

For qualitative assessment of cell viability, HUVECs were seeded in 24‐well plates at a density of 4 × 10^4^ cells per well and incubated with 1 mL of different hydrogel samples. After 24, 48, or 72 h, the supernatant was removed, and 250 µL of Calcein AM/PI working solution was added to each well. Plates were incubated for 30 min at 37°C, and fluorescence images were acquired using confocal laser scanning microscopy to visualize live (green) and dead (red) cells. This combined approach provided both quantitative and qualitative evaluation of hydrogel cytocompatibility.

### 4.15 Hemolytic Test

Hemocompatibility of HAPPF hydrogels was assessed using rabbit red blood cells (RBCs). RBCs were washed, diluted to 5% (v/v), and incubated with hydrogel extracts at 37°C for 60 min. After centrifugation, the absorbance of the supernatant at 540 nm was measured. Saline and 2% Triton X‐100 served as negative and positive controls, respectively. Hemolysis rates were calculated from triplicate measurements to evaluate the blood compatibility of the hydrogels. The hemolysis rate was calculated according to the following Equation (5):

(5) $$\begin{array}{ccc} \mathrm{Hemolysis}\;\mathrm{rate}\;(\%) & = & ({\mathrm{OD}}_{\mathrm{samples}}-{\mathrm{OD}}_{\mathrm{Saline}})/\\ & & \times \;({\mathrm{OD}}_{\mathrm{Triton}}\;X-100-\;{\mathrm{OD}}_{\mathrm{Saline}})\times 100\% \end{array}$$

where OD_samples_: the optical density of samples group, ODSaline:the optical density of negative control group, ODTriton X‐100: the optical density of positive control group.

### 4.16 Cell Migration (Scratch) Assay

HUVECs were seeded in 12‐well plates and cultured until forming a fully confluent monolayer. A 200 µL pipette tip was used to create a vertical scratch, and cells were washed twice with PBS. For the Control group, cells were incubated with serum‐free medium only, whereas the Model group was incubated with 50 µg/mL Rosup in serum‐free medium. Experimental groups were treated with 50 µg/mL Rosup in serum‐free medium containing HAPP or HAPPF hydrogel extracts. Images were captured at 0, 12, and 24 h, and the scratch area was quantified using ImageJ. Cell migration rates were calculated according to Equation (6).

(6) Scratchhealingrate(%)=At/A0×100%

where *A_t_*: scratch area at t hour, *A_0_*: scratch area at 0 h.

### 4.17 Vertical Migration

HUVECs (2 × 10^4^ cells/well) were seeded in the upper chamber of a transwell plate. For the Control group, serum‐free medium was added to the lower chamber; for the Model group, 50 µg/mL Rosup in serum‐free medium was used; experimental groups received 50 µg/mL Rosup in serum‐free medium containing HAPP or HAPPF hydrogel extracts. After 24 h incubation, cells on the upper surface were removed, and migrated cells were fixed with 4% paraformaldehyde, stained with 0.1% crystal violet for 10 min, and imaged using an upright microscope. Crystal violet was subsequently solubilized in acetic acid, and absorbance at 590 nm was measured to quantify cell migration.

### 4.18 Tube Formation Assay

Pre‐cooled 12‐well plates were coated with 30 µL Matrigel and incubated at 37°C for 30 min to allow gelation. HUVECs (8 × 10^4^ cells/well) were seeded onto the Matrigel and treated with different solutions corresponding to Control, Model, HAPP, or HAPPF groups. After 12 h incubation, tube formation was imaged using an inverted microscope. Angiogenic parameters including number of junctions, nodes, and total tube length were quantified using ImageJ.

### 4.19 In Vitro HSDG‐ NH_2_ Activation and H_2_S Release From HAPPF Hydrogels

HUVECs were seeded in 24‐well plates and cultured until reaching 50–70% confluence. Cells were then treated with the respective experimental solutions (Control, Model, HAPP, or HAPPF) and incubated for 12 h. Subsequently, the medium was replaced with fresh medium containing 10 µm CyNO_2_, a fluorescent probe for H_2_S detection, and co‐incubated for 30 min. Cells were washed three times with PBS, stained with DAPI for 5 min, and imaged using confocal laser scanning microscopy (CLSM, Zeiss LSM800) to visualize green fluorescence, representing HSDF‐ NH_2_ activation and H_2_S release.

### 4.20 In Vitro Evaluation of Hydrogel‐Mediated ROS Scavenging in HUVECs

HUVECs (1 × 10^5^ cells/well) were seeded in 24‐well plates. After adhesion, cells were treated with different solutions corresponding to Control, Model, HAPP, or HAPPF groups and incubated for 12 h. Cells were then washed, stained with 10 µm DHE for 30 min, counterstained with DAPI, and imaged by CLSM (Zeiss LSM800) to evaluate intracellular ROS and the antioxidant effects of the hydrogels.

### 4.21 Immunofluorescence Staining

HUVECs were seeded in µ‐slide 8‐well plates and treated for 24 h according to four groups: Control, Model, HAPP, or HAPPF. After treatment, cells were fixed with 2% paraformaldehyde, permeabilized with 0.1% Triton X‐100 for 10 min, and blocked with 1% BSA for 30 min. Cells were incubated overnight at 4°C with primary antibodies against VEGF, NGF, CGRP, or TGF‐β1, followed by 1 h incubation at room temperature with fluorescent secondary antibodies. Nuclei were counterstained with DAPI for 5 min. Confocal laser scanning microscopy (CLSM, Zeiss LSM800) was used to acquire images and evaluate the immunomodulatory, neurogenic, and pro‐angiogenic effects of the hydrogels under oxidative stress.

### 4.22 Quantification of Inflammatory Cytokines in HUVECs

HUVECs were seeded in 24‐well plates at 1 × 10^5^ cells/well and cultured to 50%–70% confluence. Cells were treated according to four groups: Control, Model, HAPP, or HAPPF, with the Model, HAPP, and HAPPF groups containing 50 µg/mL Rosup. After 24 h incubation, culture supernatants were collected and centrifuged to remove debris. TNF‐α, IL‐6, and IL‐1β levels were measured using commercial ELISA kits following the manufacturers’ protocols. Absorbance was read at 450 nm, and cytokine concentrations were calculated based on standard curves.

### 4.23 Animals

All animal procedures were approved by the Experimental Animal Ethics Committee of Jinan University (Approval No. 20200314‐07) and were conducted in accordance with the relevant guidelines and regulations. A total of 80 male Sprague‐Dawley rats (6‐8 weeks old, 180–210 g) and 10 male New Zealand white rabbits (4 weeks old, ≈2.5 kg) were obtained from the Guangdong Medical Laboratory Animal Center (Guangzhou, China). All animals were housed under specific pathogen‐free (SPF) conditions with a controlled temperature (22 ± 1°C) and a 12/12 h light/dark cycle, and provided with ad libitum access to food and water. Animal health was monitored daily, and no adverse events were reported throughout the study.

### 4.24 Full‐Thickness Wound Model and Treatment in Diabetic Rats

Forty male SD rats were used. Diabetes was induced by a single intraperitoneal injection of streptozotocin (STZ, 60 mg/kg) in 0.1 m citrate buffer (pH *4.5*). Blood glucose was measured 72 h post‐injection, and rats with fasting glucose ≥ 16.7 mmol/L were considered diabetic. After 14 days of stabilization, full‐thickness circular wounds (10 mm diameter) were created on the dorsum under isoflurane anesthesia (1.5 L/min) following shaving and disinfection with 75% ethanol. Rats were randomly assigned to five groups: Normal (non‐diabetic control), Model (diabetic control), 3M Tegaderm (positive control), HAPP, and HAPPF. The wounds in the 3M Tegaderm, HAPP, and HAPPF groups were covered with 3M Tegaderm to secure the dressing. Rats were returned to their cages for free access to food and water after awakening. Dressings were replaced every 2 days. Wound healing was documented by photographing at 0, 3, 7, 9, 11, and 14 days. Wound areas were quantified using ImageJ, and the wound contraction rate was calculated as Equation (7):

(7) $$Wound\;contraction\;rate\;\left(\%\right)=\left(A0\;-\;At\right)\;/\;A0\;\times 100\%\;$$

where *A*0: the wound area at 0 day; *A*t :the wound area at t day.

### 4.25 In Vivo Spatiotemporal Monitoring and Quantification of H_2_S Release

To systematically evaluate the ROS‐responsive activation of the donor and subsequent H_2_S release, we integrated real‐time fluorescence imaging with biochemical quantification. Diabetic male SD rats were randomly assigned to two imaging cohorts (n = 3 per group): (1) the HAPPF group, treated with the HSDF‐NH_2_ donor‐loaded hydrogel to track its intrinsic fluorescence transition upon ROS‐triggered activation, and (2) the HAPPF + Cy‐NO_2_ group, treated with a hydrogel co‐loaded with the donor and a specific probe (Cy‐NO_2_, 100 µm) to detect liberated H_2_S. Following the creation of full‐thickness wounds (10 mm diameter), the hydrogels were applied, and fluorescence signals were captured at multiple channels using an in vivo imaging system (IVIS). Consistency was maintained across all time points by using identical exposure and gain settings.To provide rigorous quantitative validation of the imaging data, H_2_S concentrations within the wound site were further measured using a Hydrogen Sulfide Assay Kit (Shanghai Keaibo, China). Skin tissue homogenates were prepared at predetermined intervals, and the H_2_S content in the supernatants was quantified and normalized to the total protein concentration (determined by BCA assay). By combining real‐time spatiotemporal imaging with precise biochemical analysis, the in vivo release kinetics of H_2_S were comprehensively characterized.

### 4.26 Diabetic Rabbit Ear Hypertrophic Scar Model

Ten healthy New Zealand white rabbits were acclimated for 1 week and fasted for 12 h (water ad libitum) before baseline fasting glucose was measured (< 6.0 mmol/L). Rabbits were randomly assigned to Control, Diabetic Model, Positive Control, HAPP, or HAPPF groups. To induce diabetes, alloxan monohydrate (Alloxan, 100 mg/kg, dissolved in 0.9% sterile saline) was administered via a rapid ear marginal intravenous injection, while the Control group received an equivalent volume of saline. Blood glucose levels were measured 48 h post‐injection. Animals that did not meet the diabetic criteria (≥ 14 mmol/L once or ≥ 11 mmol/L twice) received an additional dose of 50 mg/kg alloxan. Blood glucose was monitored weekly for 2 weeks to ensure the stability of the diabetic model. Full‐thickness ear wounds were created and treated according to group assignments. Wounds were covered with sterile dressings, replaced every 3 days, and wound healing was monitored via photography. On day 30, three independent evaluators (blinded to the groups) scored scar formation using the Vancouver Scar Scale (VSS). Following humane euthanasia, skin samples including the attached cartilage were collected and fixed in 4% paraformaldehyde for histological analysis.

### 4.27 Histological, Immunofluorescence, and Immunohistochemical Analyses

Wound tissue samples from diabetic rats and rabbit ear scar samples (day 30) were collected and fixed in 4% paraformaldehyde (*n* = 6 independent biological replicates per group). Rabbit ear samples underwent an additional decalcification process in 10% EDTA for 3 weeks. After paraffin embedding and sectioning, H&E, Masson's Trichrome, and Sirius Red staining were performed. The wound and scar areas were strictly defined as the regions between the original wound margins, characterized by the transition in tissue architecture and the absence of skin appendages.

To ensure objectivity and eliminate observer bias, all image acquisition and quantitative analyses were conducted in a blinded manner by two independent researchers. Immunofluorescence (DHE, CD86, CD206, VEGF, NGF, and CGRP) and immunohistochemistry (TNF‐α, TGF‐β1, α‐SMA, p‐Smad2, p‐Smad3) were performed following standard protocols. For each section, three to five non‐overlapping regions of interest (ROIs) within the wound/scar bed were randomly selected. All images were captured using a digital slide scanner (Panoramic MIDI, 3DHISTECH, Hungary). To compensate for potential variability in section depth and tissue density, the fluorescence and staining intensities were quantified as integrated optical density (IOD) or mean fluorescence intensity (MFI) and subsequently normalized to the tissue area or DAPI‐positive nuclei count. Quantitative assessments, including scar thickness, SEI, CVF, and positive areas, were performed using ImageJ software based on six parallel samples per group.

### 4.28 Quantitative Analysis of Cytokines and Signaling Molecules

The concentrations of cytokines and signaling molecules in cell culture supernatants and skin tissues were quantified using commercial ELISA kits according to the manufacturer's instructions. For tissue analysis, rabbit ear skin samples (*n* = 6 per group) were harvested and homogenized in pre‐chilled PBS containing protease inhibitors. The wound area was strictly defined as the region between the original wound margins, characterized by the transition of tissue architecture and absence of skin appendages. To ensure unbiased evaluation, the collection of tissue homogenates and all subsequent assays were performed in a blinded manner. To compensate for potential biological and technical variability, total protein concentrations in the supernatants were determined using a BCA protein assay kit, and all ELISA data were normalized to the total protein content. Absorbance was measured at 450 nm using a microplate reader in triplicate for each sample, and results were calculated based on standard curves.

### 4.29 In Vivo Safety Evaluation

On day 14, rats were euthanized via intraperitoneal injection of sodium pentobarbital (150 mg/kg). Major organs, including heart, liver, spleen, lung, and kidney, were harvested and fixed in 4% paraformaldehyde for 24 h. After dehydration, tissues were embedded in paraffin, sectioned, and subjected to H&E staining. Morphological changes were examined using an Olympus BX‐53 microscope to assess potential histopathological alterations induced by HAPPF hydrogels. Concurrently, blood samples were collected into EDTA‐K2 anticoagulant tubes, gently mixed, and analyzed for hematological parameters using an automated biochemical analyzer to evaluate systemic biocompatibility.

### 4.30 RNA Sequencing and Transcriptomic Analysis

Total RNA was extracted from wound tissue samples of diabetic rat models and prepared for cDNA synthesis following standard protocols. RNA sequencing was performed by Origingene Biomedical Technology Co., Ltd. (Shanghai, China). The resulting cDNA libraries were sequenced on an Illumina HiSeq X‐Ten platform (LC Bio, China). Raw sequencing reads were evaluated using FastQC v0.11.4, and low‐quality reads and adaptors were removed to obtain clean reads. Clean reads were aligned to the rat reference genome (Rnor_6.0) using HISAT2. Gene expression levels were quantified as reads per kilobase per million mapped reads (RPKM). Differentially expressed genes (DEGs) were identified based on fold change >1 and false discovery rate (FDR) < 0.05. Gene Ontology (GO) enrichment analysis was subsequently performed using singular enrichment analysis to explore functional categories associated with wound healing and the inhibition of scar‐related transcriptional changes.

### 4.31 Statistical Analysis

All quantitative data are expressed as mean ± standard deviation (SD) from at least three independent experiments. Data pre‐processing, including the assessment of normality (Shapiro‐Wilk test) and homogeneity of variance (Levene's test), was performed prior to statistical comparison. No outliers were excluded from the analyses. Statistical significance between multiple groups was assessed using one‐way analysis of variance (ANOVA) followed by Tukey's post hoc test for multiple comparisons. All statistical tests were two‐sided and conducted using SPSS 27.0 (SPSS Inc., USA). The specific sample size (n) for each experimental group, data presentation details, and statistical significance levels are clearly indicated in the corresponding figure legends. *P* values less than 0.05 were considered statistically significant (^*^ *p* < 0.05, ^**^ *p* < 0.01, ^***^ *p* < 0.001).

## Author Contributions

X.Y.N., Z.Q.Z., B.M.L., H.L., H.Y.W. and Z.L.H. performed the experiments. X.Y.N., Z.Q.Z., and B.M.L. analyzed the data and drafted the manuscript. P. H. and X.Y.N. designed the study. G.L. and P.H. reviewed and edited the manuscript. All authors read and approved the final version of the manuscript.

## Funding

This work was supported by the National Natural Science Foundation of China (Grant No. 82071367), the Natural Science Foundation of Guangdong Province (Grant No. 2023A1515011135) the Guangzhou Clinical Characteristic Technology Project (Grant No. 2023C‐TS46), and the Guangzhou Science and Technology Project (Grant Nos. 2025A03J3601 and 2024A03J0665).

## Conflicts of Interest

The authors declare no conflicts of interest.

## Supporting information




**Supporting File**: advs76337‐sup‐0001‐SuppMat.docx.

## Data Availability

The data that support the findings of this study are available from the corresponding author upon reasonable request.
