# Supplementary material for: A Hydrogen Sulfide–Releasing Dynamic Hydrogel Modulates Coordinated Neurovascular, Immune, and Angiogenic Responses for Scar‐Suppressed Diabetic Wound Repair
Source: Adv Sci (Weinh). 2026 Jun 27;13(53):e76337. doi: 10.1002/advs.76337 (PMC13336870; doi:10.1002/advs.76337)
Supplement: Supplementary file 1 [file ADVS-13-e76337-s001.docx]

Supporting Information

**A hydrogen sulfide–releasing dynamic hydrogel modulates coordinated neurovascular, immune, and angiogenic responses for scar-suppressed diabetic wound repair**

Xuyang Ning^a, b ,c#^,Ziqiang Zhou^a, b, c#^, Bangming Li^d,a#^, Hong Lu^b,c^, Haoyang Wen^b,c^,Zhoulong Huang^a^,Gang Li^a*^, Ping Hu ^a, b, c*^

a Department of Burns & Plastic Surgery, Guangzhou Red Cross Hospital, Faculty of Medical Science, Jinan University, Guangzhou 510006, China.

b State Key Laboratory of Bioactive Molecules and Druggability Assessment, Jinan University, Guangzhou 510006, China.

c College of Pharmacy, Jinan University, Guangzhou 510006, China.

d First Clinical Medical College, Guangdong Medical University, Zhanjiang, 524000,China.

#: These authors contributed equally.

Corresponding author: Gang Li, Ping Hu

Mailing address: Jinan University, No.855, East Xingye Avenue, Panyu Districst, Guangzhou 510632, CHINA

Tel.: +86-18581483142

E-mail: 182340409@qq.com, inzahu@hotmail.com

**Content**

**Supplementary Experimental 1: In Vivo Injectability, Rapid Degradation, and Tissue Biocompatibility.**

**Supplementary Experimental 2: In Situ Detection of H_2_S and Donor Activation in Skin Tissues.**

**Figure S1.** HAPP structure recovery scan with different proportions.

**Figure S2.** Comparative H₂O₂ scavenging efficiency of HAPP and HAPPF hydrogels at 1 h and 12 h.

**Figure S3.** Live/dead staining images of HUVECs cultured with different hydrogel formulations after 48 h under oxidative stress.

**Figure S4.** Representative images of crystal violet staining in HUVECs under different treatments.

**Figure S5.** Representative images of the tube formation assay in HUVECs.

**Figure S6.** Semi-quantitative analysis of the number of junctions and meshes formed by HUVECs cultured with different hydrogel extracts.

**Figure S7.** Representative fluorescence images showing ROS-stimulated autofluorescence of HSDF-NH₂ and H₂S-specific fluorescence detected by Cy-NO_2_ probe.

**Figure S8.** Representative images of intracellular ROS levels in HUVECs for different groups(20×,scale bar: 20 μm).

**Figure S9.** Representative immunofluorescence images showing VEGF expression in HUVECs under different treatment groups.

**Figure S10.** Representative immunofluorescence images showing NGF expression in HUVECs under different treatment groups.

**Figure S11.** Representative immunofluorescence images showing CGRP expression in HUVECs under different treatment groups.

**Figure S12.** Representative immunofluorescence images showing TGF-β expression in HUVECs under different treatment groups.

**Figure S13.** Semi-quantitative analysis of relative fluorescence intensities for VEGF (A), NGF (B), CGRP (C), and TGF-β (D) expression in HUVECs under different treatment groups.

**Figure S14.** Representative fluorescence images of skin tissue sections at different time points post-treatment. The green fluorescence represents the distribution of the H_2_S donor (HSDG-NH_2_), while the red/magenta fluorescence indicates the presence of H_2_S as detected by the specific probe (CY-NO_2_) within the wound tissue.

**Figure S15.** Quantitative analysis of re-epithelialization rates across different treatment groups at various time points.

**Figure S16.** Quantitative analysis of collagen volume fraction (CVF) across different treatment groups at various time point.

**Figure S17.** Representative immunofluorescence images of wound sections at day 7 and day 14 post-treatment.

**Figure S18.** Macrophage polarization in diabetic wounds.Representative immunofluorescence images showing macrophage phenotypes.

**Figure S19.** Angiogenesis in diabetic wounds. Representative immunofluorescence images of wound sections showing VEGF expression.

**Figure S20.** ELISA quantification of vascular endothelial growth factor (VEGF) concentrations in skin tissues from different treatment groups on days 7 and 14 post-surgery.

**Figure S21.** Evaluation of nerve regeneration in diabetic wounds. Representative immunofluorescence images of wound sections on day 14 post-treatment.

**Figure S22.** ELISA quantification of NGF and CGRP concentrations in wound homogenates on day 14 post-treatment.

**Figure S23.** Quantitative analysis of the relative expression levels of TGF-β.

**Figure S24.** ELISA quantification of TNF-α levels in skin tissues from different treatment groups on days 7 and 14 post-surgery.

**Figure S25.** ELISA quantification ofTGF-β concentrations in skin tissue homogenates on days 7 and 14 post-treatment.

**Figure S26.** In vivo degradation profile of the HAPPF hydrogel.(A) Schematic illustration of the subcutaneous implantation procedure in a rat model. (B) In vivo degradation kinetics of the HAPPF hydrogel, expressed as the percentage of residual weight at indicated time points.

**Figure S27.** Histocompatibility evaluation of the HAPPF hydrogel.

**Figure S28.** Bubble plot of 21 hematological parameters across different treatment groups.

**Figure S29.** H&E staining of major organs (heart, liver, spleen, lung, and kidney) from different treatment groups to evaluate in vivo biocompatibility.

**Figure S30.** DEG statistics and distribution analysis. (A) Number of up- and down-regulated DEGs in comparison group. (B–D) Volcano plots showing DEG distribution for (B) HAPP vs. Model, (C) HAPPF vs. Model, and (D) HAPPF vs. HAPP.

**Figure S31.** GSEA enrichment analysis of core signaling pathways in diabetic wound repair. (A-C) Representative GSEA plots showing enriched pathways for (A) HAPP vs. Model, (B) HAPPF vs. Model, and (C) HAPPF vs. HAPP.

**Figure S32.** Global GO enrichment analysis of DEGs across comparison groups. Representative GO enrichment terms categorized by biological process (BP), molecular function (MF), and cellular component (CC) for HAPP vs. Model, HAPPF vs. Model, and HAPPF vs. HAPP groups.

**Figure S33.** ELISA quantification of biochemical markers in skin tissues on day 14. Concentrations of (A) PPARγ, (B) iNOS, (C) BDNF, and (D) IL-17A in skin tissue homogenates across experimental groups.

**Figure S34.** Semi-quantitative analysis of CVF in rabbit ear scar tissues at day 30.

**Figure S35.** Semi-quantitative analysis of Type I/III collagen ratio in rabbit ear scar tissues at day 30.

**Figure S36.** Semi-quantitative immunohistochemical analysis of TGF-β/Smad signaling pathway markers in rabbit ear scar tissues at day 30.(A) Relative protein expression levels of p-Smad3 across all groups. (B) Relative protein expression levels of p-Smad2.

**Supplementary Experimental**

**1. In Vivo Injectability, Rapid Degradation, and Tissue Biocompatibility.**

The in vivo performance of the HAPPF hydrogel was evaluated using a subcutaneous injection model in SD rats (*n* = 3). Briefly, 400 μL of the HAPPF was subcutaneously injected into the dorsal area using a 1-mL syringe with a 21-gauge needle. To monitor the initial degradation kinetics, the gross morphology of the injected hydrogel was photographed, and the remaining mass was quantified at 0, 2, 4, 8, 12, and 24 h post-injection. The harvested samples were gently cleaned, lyophilized, and weighed. The remaining weight percentage was calculated relative to the initial mass (W_0_) to generate a degradation profile.

To further assess long-term tissue response and material-tissue integration, skin samples at the injection site were collected at 0, 7, 14, and 28 days for histological evaluation. The harvested tissues were fixed in 4% paraformaldehyde, embedded in paraffin, and sectioned for H&E staining. Images were captured at 5× and 40× magnification using a digital slide scanner to evaluate inflammatory cell infiltration, collagen remodeling, and the gradual replacement of the hydrogel by host tissue over the 28-day period.

**2. In Situ Detection of H_2_S and Donor Activation in Skin Tissues.**

To visualize the dynamic H_2_S release and ROS-responsive donor activation, skin tissues collected from the wound/scar bed (treated with the HAPPF+HSDF-NH_2_+Cy-NO_2_ hydrogel, $n = 3) were processed for cryosectioning and fluorescence imaging.Briefly, at predetermined time points (2, 4, 8, 12, 24, and 48 h), harvested tissues were fixed in 4% paraformaldehyde, cryoprotected in 30% sucrose, and embedded in OCT compound. Serial cryosections (10–15 μm thick) were prepared. To detect H_2_S in situ, the sections were stained with a specific fluorescent probe, Cy-NO_2_ (100 μM, red channel), which is triggered upon H_2_S detection. Simultaneously, the intrinsic green fluorescence of the HSDF-NH_2_ donor (green channel), activated via ROS response, was monitored.Fluorescence images were acquired using a digital slide scanner (Panoramic MIDI, 3DHISTECH) under consistent exposure times and gain settings across all time points. The spatiotemporal distribution of liberated H_2_S and the donor's activation state were tracked by overlaying the Cy-NO_2_ (red), activated HSDF-NH_2_ (green), and DAPI-counterstained nuclei (blue) channels.

**Figure S1.** HAPP structure recovery scan with different proportions.

**Figure S2.** Comparative H₂O₂ scavenging efficiency of HAPP and HAPPF hydrogels at 1 h and 12 h(n = 6 independent samples).

**Figure S3.** Live/dead staining images of HUVECs cultured with different hydrogel formulations after 48 h under oxidative stress（Scale bar: 200 μm）.

**Figure S4.** Representative images of crystal violet staining in HUVECs under different treatments(n = 3 independent samples, scale bar: 200 μm).

**Figure S5.** Representative images of the tube formation assay in HUVECs(n = 3 independent samples, scale bar: 100 μm).

**Figure S6.** Semi-quantitative analysis of the number of junctions and meshes formed by HUVECs cultured with different hydrogel extracts(*n* = 3 independent samples, **P* < 0.05, ***P* < 0.01, ****P* < 0.001 by one-way ANOVA followed by Tukey's post hoc test).

**Figure S7.** Representative fluorescence images showing ROS-stimulated autofluorescence of HSDF-NH_2_ and H_2_S-specific fluorescence detected by Cy-NO_2_ probe (20×,scale bar: 20 μm).

**Figure S8.** Representative images of intracellular ROS levels in HUVECs for different groups(20×,scale bar: 20 μm).

**Figure S9.** Representative immunofluorescence images showing VEGF expression in HUVECs under different treatment groups (60×,scale bar: 5 μm).

**Figure S10.** Representative immunofluorescence images showing NGF expression in HUVECs under different treatment groups (60×,scale bar: 5 μm).

**Figure S11.** Representative immunofluorescence images showing CGRP expression in HUVECs under different treatment groups (60×,scale bar: 5 μm).

**Figure S12.** Representative immunofluorescence images showing TGF-β expression in HUVECs under different treatment groups (60×,scale bar: 5 μm).

**Figure S13.** Semi-quantitative analysis of relative fluorescence intensities for VEGF (A), NGF (B), CGRP (C), and TGF-β (D) expression in HUVECs under different treatment groups (*n* = 3 independent samples, **P* < 0.05, ***P* < 0.01, ****P* < 0.001 by one-way ANOVA followed by Tukey's post hoc test).

**Figure S14.** Representative fluorescence images of skin tissue sections at different time points post-treatment. The green fluorescence represents the distribution of the H_2_S donor (HSDG-NH₂), while the red/magenta fluorescence indicates the presence of H_2_S as detected by the specific probe (CY-NO_2_) within the wound tissue (Scale bar: 100 μm).

**Figure S15.** Quantitative analysis of re-epithelialization rates across different treatment groups at various time points(*n* = 6 independent samples, **P* < 0.05, ****P* < 0.001, *****P* < 0.0001 by one-way ANOVA followed by Tukey's post hoc test).

**Figure S16.** Quantitative analysis of collagen volume fraction (CVF) across different treatment groups at various time points(*n* = 6 independent samples, **P* < 0.05, ***P* < 0.01, ****P* < 0.001, *****P* < 0.0001 by one-way ANOVA followed by Tukey's post hoc test).

**Figure S17.** Representative immunofluorescence images of wound sections at day 7 and day 14 post-treatment. Red fluorescence (DHE staining) represents ROS levels, green fluorescence indicates the distribution of the H_2_S donor (HSDG-NH_2_), and blue fluorescence (DAPI) labels the cell nuclei. Top rows: 5× magnification (Scale bar: 1000 μm); Bottom rows: 20× magnification (Scale bar: 200 μm).

**Figure S18.** Macrophage polarization in diabetic wounds.Representative immunofluorescence images showing macrophage phenotypes. Blue: DAPI; Red: CD86 (M1); Green: CD206 (M2). Merged images display 5× (Scale bar: 1000 μm) and 20× (Scale bar: 200 μm) magnifications.

**Figure S19.** Angiogenesis in diabetic wounds. Representative immunofluorescence images of wound sections showing VEGF expression. Green: VEGF; Blue: Nuclei (DAPI). Merged images display 5× (Scale bar: 1000 μm) and 20× (Scale bar: 200 μm) magnifications on days 7 and 14 post-treatment.

**Figure S20.** ELISA quantification of vascular endothelial growth factor (VEGF) concentrations in skin tissues from different treatment groups on days 7 and 14 post-surgery(*n* = 6 independent samples, **P* < 0.05, ***P* < 0.01, ****P* < 0.001 by one-way ANOVA followed by Tukey's post hoc test).

**Figure S21.** Evaluation of nerve regeneration in diabetic wounds. Representative immunofluorescence images of wound sections on day 14 post-treatment. Red: NGF; Green: CGRP; Blue: Nuclei (DAPI). Merged images display 5× (Scale bar: 1000 μm) and 20× (Scale bar: 200 μm) magnifications.

**Figure S22.** ELISA quantification of NGF and CGRP concentrations in wound homogenates on day 14 post-treatment(*n* = 6 independent samples, **P* < 0.05, ***P* < 0.01, ****P* < 0.001 by one-way ANOVA followed by Tukey's post hoc test).

**Figure S23.** Quantitative analysis of the relative expression levels of TGF-β (*n* = 6 independent samples, **P* < 0.05, ***P* < 0.01, ****P* < 0.001 by one-way ANOVA followed by Tukey's post hoc test).

**Figure S24.** ELISA quantification of TNF-α levels in skin tissues from different treatment groups on days 7 and 14 post-surgery(*n* = 6 independent samples, **P* < 0.05, ***P* < 0.01, ****P* < 0.001 by one-way ANOVA followed by Tukey's post hoc test).

**Figure S25.** ELISA quantification ofTGF-β concentrations in skin tissue homogenates on days 7 and 14 post-treatment(*n* = 6 independent samples, **P* < 0.05, ***P* < 0.01, ****P* < 0.001 by one-way ANOVA followed by Tukey's post hoc test).

**Figure S26.** In vivo degradation profile of the HAPPF hydrogel.

(A) Schematic illustration of the subcutaneous implantation procedure in a rat model. (B) In vivo degradation kinetics of the HAPPF hydrogel, expressed as the percentage of residual weight at indicated time points (n = 3 independent samples).

**Figure S27.** Histocompatibility evaluation of the HAPPF hydrogel.

Representative H&E-stained images of the skin tissues surrounding the HAPPF implants at indicated time points post-implantation.

**Figure S28.** Bubble plot of 21 hematological parameters across different treatment groups.

The plot compares 21 blood routine parameters, including WBC, RBC, HGB, and PLT, among the various groups, illustrating no significant systemic toxicity. Bubble size and color represent the relative values of each parameter.

**Figure S29.** H&E staining of major organs (heart, liver, spleen, lung, and kidney) from different treatment groups to evaluate in vivo biocompatibility.

Representative histological images show no observable tissue damage or abnormalities among groups, indicating that the treatments are biocompatible (scale bar: 500 μm).

**Figure S30.** DEG statistics and distribution analysis. (A) Number of up- and down-regulated DEGs in comparison groups (|FC| > 2, FDR < 0.05). (B–D) Volcano plots showing DEG distribution for (B) HAPP vs. Model, (C) HAPPF vs. Model, and (D) HAPPF vs. HAPP (|log_2_FC| > 1.5, FDR < 0.05). Red/blue dots indicate significantly up-/down-regulated genes; grey dots represent non-significant (NS) genes(n = 3 independent samples).

**Figure S31.** GSEA enrichment analysis of core signaling pathways in diabetic wound repair. (A-C) Representative GSEA plots showing enriched pathways for (A) HAPP vs. Model, (B) HAPPF vs. Model, and (C) HAPPF vs. HAPP. Key pathways include PPAR, NF-κB, and TGF-β signaling, illustrating the regulatory effects of HAPP and HAPPF on the wound microenvironment at the transcriptomic level(n = 3 independent samples).

**Figure S32.** Global GO enrichment analysis of DEGs across comparison groups. Representative GO enrichment terms categorized by biological process (BP), molecular function (MF), and cellular component (CC) for HAPP vs. Model, HAPPF vs. Model, and HAPPF vs. HAPP groups. The HAPPF group exhibits significant enrichment in lipid metabolic processes and muscle contraction-related pathways, indicating its superior capacity in promoting functional tissue reconstruction and metabolic reprogramming in diabetic wounds(n = 3 independent samples).

**Figure S33.** ELISA quantification of biochemical markers in skin tissues on day 14. Concentrations of (A) PPARγ, (B) iNOS, (C) BDNF, and (D) IL-17A in skin tissue homogenates across experimental groups. The results highlight the modulatory effects of HAPPF on metabolic, neurotrophic, and inflammatory pathways(*n* = 6 independent samples, **P* < 0.05, ***P* < 0.01, ****P* < 0.001 by one-way ANOVA followed by Tukey's post hoc test).

**Figure S34.** Semi-quantitative analysis of CVF in rabbit ear scar tissues at day 30(*n* = 6 independent samples, **P* < 0.05, ***P* < 0.01, ****P* < 0.001 by one-way ANOVA followed by Tukey's post hoc test).

**Figure S35.** Semi-quantitative analysis of Type I/III collagen ratio in rabbit ear scar tissues at day 30(*n* = 6 independent samples, **P* < 0.05, ***P* < 0.01, ****P* < 0.001 by one-way ANOVA followed by Tukey's post hoc test).

**Figure S36.** Semi-quantitative immunohistochemical analysis of TGF-β/Smad signaling pathway markers in rabbit ear scar tissues at day 30.(A) Relative protein expression levels of p-Smad3 across all groups. (B) Relative protein expression levels of p-Smad2 (*n* = 6 independent samples, **P* < 0.05, ***P* < 0.01, ****P* < 0.001 by one-way ANOVA followed by Tukey's post hoc test).
